# Formation of Nudicaulins In Vivo and In Vitro and the Biomimetic Synthesis and Bioactivity of *O*-Methylated Nudicaulin Derivatives

**DOI:** 10.3390/molecules23123357

**Published:** 2018-12-18

**Authors:** Bettina Dudek, Florian Schnurrer, Hans-Martin Dahse, Christian Paetz, Anne-Christin Warskulat, Christiane Weigel, Kerstin Voigt, Bernd Schneider

**Affiliations:** 1 Max Planck Institute for Chemical Ecology, Hans-Knöll-Str. 8, D-07745 Jena, Germany; bdudek@ice.mpg.de (B.D.); fschnurrer@ice.mpg.de (F.S.); cpaetz@ice.mpg.de (C.P.); anne.warskulat@yahoo.de (A.-C.W.); 2 Leibniz Institute for Natural Product Research and Infection Biology, Hans Knöll Institute (HKI), Adolf-Reichwein-Straße 23, D-07745 Jena, Germany; hans-martin.dahse@hki-jena.de (H.-M.D.); christiane.weigel@hki-jena.de (C.W.); kerstin.voigt@hki-jena.de (K.V.)

**Keywords:** nudicaulins, flower pigments, indole alkaloids, biomimetic synthesis, bioassays

## Abstract

Nudicaulins are yellow flower pigments accounting for the color of the petals of *Papaver nudicaule* (Papaveraceae). These glucosidic compounds belong to the small group of indole/flavonoid hybrid alkaloids. Here we describe in vivo and in vitro experiments which substantiate the strongly pH-dependent conversion of pelargonidin glucosides to nudicaulins as the final biosynthetic step of these alkaloids. Furthermore, we report the first synthesis of nudicaulin aglycon derivatives, starting with quercetin and ending up at the biomimetic fusion of a permethylated anthocyanidin with indole. A small library of nudicaulin derivatives with differently substituted indole units was prepared, and the antimicrobial, antiproliferative and cell toxicity data of the new compounds were determined. The synthetic procedure is considered suitable for preparing nudicaulin derivatives which are structurally modified in the indole and/or the polyphenolic part of the molecule and may have optimized pharmacological activities.

## 1. Introduction

**Figure 1:**
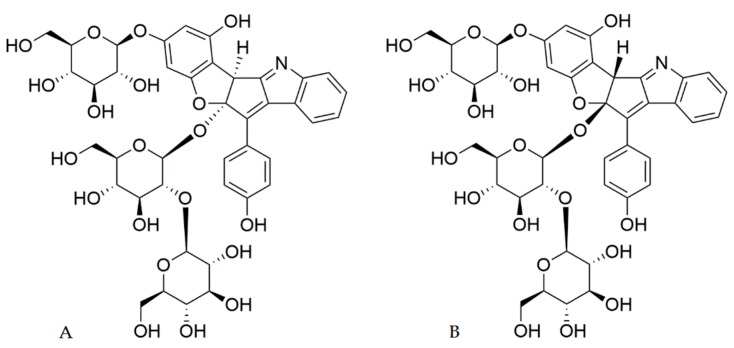
Structures of nudicaulin I (**A**) and II (**B**), flower pigments of *Papaver nudicaule* L.

The yellow pigment of *Papaver nudicaule* L. (“Iceland poppy”) petals was first reported in 1939 and named nudicaulin [1]. In 2006, eight differently substituted compounds, also named nudicaulins, with two stereoisomeric aglycons were identified from petals of the yellow blooming cultivar of this plant. An indole/flavonoid hybrid structure was proposed for these alkaloids in 2006 [2], and the revised constitution and the absolute configuration (Figure 1) were reported in 2013 [3]. The relative and absolute configurations of nudicaulins (Figure 1) were assigned by NOESY spectroscopy and by comparison of the experimental CD spectra with calculated spectra, respectively [3].

Biosynthetic studies [4,5] revealed pelargonidin glucosides and indole as probable biosynthetic precursors as well as the building blocks of these unique polyphenolic molecules (Scheme S1). A mechanism for the formation of the nudicaulins was proposed [5], but the conditions or enzymes necessary to enable the reaction in the plant remained unknown. In the first part of the present study, we therefore aimed at shedding light on the final step of the nudicaulin biosynthesis. Our in vivo and in vitro experiments provide evidence that the proposed precursors are indeed used by *P. nudicaule* and that their fusion is possible without enzymatic catalysis.

Nudicaulins can be considered flavonoidal indole alkaloids. Compared to the large class of monoterpenoidal indole alkaloids, the flavonoidal indole alkaloids are a small group with only a few known compounds, such as lotthanongine [6] and yuremamine [7,8]. Nevertheless, because of the immense pharmacological importance of some indole alkaloids, e.g., the anticancer drugs vincristine and vinblastine [9,10], we aimed at the development of a synthesis of nudicaulin aglycon derivatives and investigated their bioactivity.

## 2. Results

### 2.1. The Final Step of the Nudicaulin Biosynthesis

**Figure 2:**
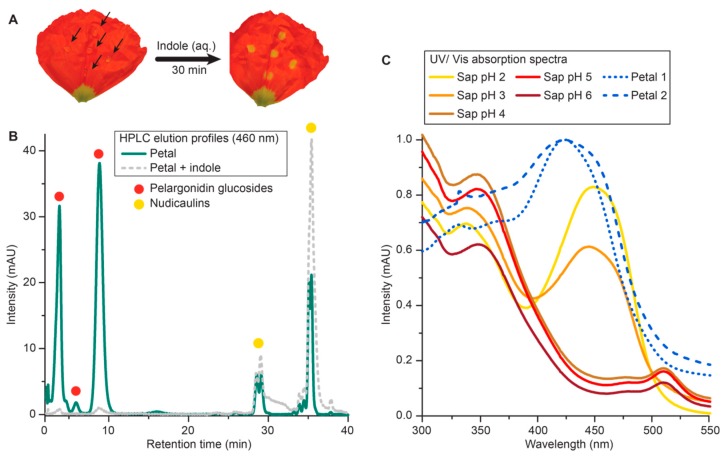
(**A**) In vivo formation of nudicaulins: Drops of aqueous indole solution (1 mg·mL^−1^) were applied to a petal of the orange *P. nudicaule* cultivar. After 30 min, the color of the treated petal area changed to yellow. (**B**) HPLC elution profile (460 nm, HPLC 1, method 1) of a petal extract of the orange *P. nudicaule* cultivar (dark green line) compared with the elution profile of an extract of a petal which had been immersed in indole solution for 20 min (gray line). Pelargonidin glucosides ● were transformed into nudicaulins ● during treatment with indole solution. (**C**) UV/Vis absorption spectra of the cell sap of yellow *P. nudicaule* petals at different pH values (solid lines) in comparison with the spectra of intact petals (dotted lines). The typical absorption in the spectral region around 450 nm which accounts for the yellow color is present only in strongly acidic cell sap.

An in vivo experiment using a petal of the orange *P. nudicaule* cultivar was carried out to prove that the free indole is incorporated into the nudicaulin molecule. In orange petals, a mixture of red pelargonidin glucosides and yellow nudicaulins is responsible for the color. Under natural conditions, the low level of indole [11] probably confines the conversion of an excess of pelargonidin glucosides into nudicaulins. Therefore, drops of an aqueous indole solution were applied to the surface of orange petals. After approximately 30 min, the area covered by the indole solution had turned yellow while the untreated petal surface remained orange (Figure 2A). Treating a whole petal with indole solution for 20 min, followed by extraction, HPLC analysis and comparison with the extract of an untreated petal, clearly showed the disappearance of pelargonidin glucosides and the formation of nudicaulins (Figure 2B). This result showed that free indole and pelargonidin glucosides are indeed the final precursors of nudicaulins.

Additionally, we aimed to perform in vitro experiments. Preliminary investigations indicated that the color of petal extracts depended on their pH value. The UV/Vis absorption of cell sap from yellow *P. nudicaule* petals at pH values 4 and 3 differed completely. At a pH value higher than or equal to 4, spectra show a strong maximum at 348 nm and a weak maximum at 510 nm. If the pH value is lower than or equal to 3, the maxima are around 450 and 337 nm. In comparison, the absorption spectrum of a petal measured under in vivo conditions has a broad maximum around 422 nm and two shoulders at 331 and 360 nm (Figure 2C). The shoulders originate mainly from kaempferol glucosides, which are also present in high amounts in the petals [2]. The absorption in the blue region is much more intense than the absorptions at shorter wavelengths. In the in vitro experiment, this difference holds true only for a pH of 2, suggesting that the vacuoles where the pigments are stored possess an unusually acidic pH value [12], probably equal to or lower than pH 2.

**Figure 3:**
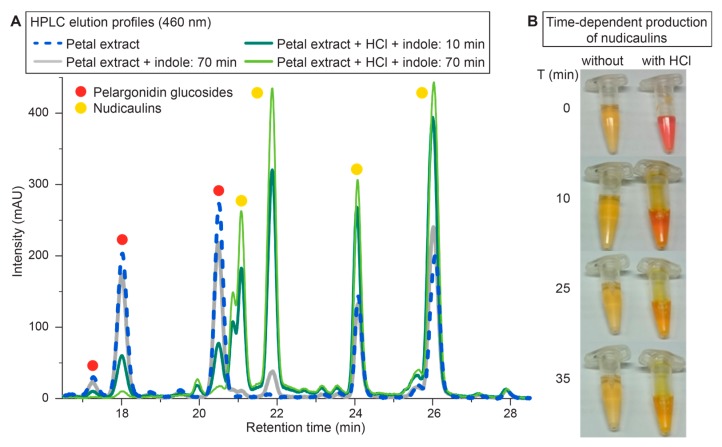
In vitro formation of nudicaulins in an extract of orange *P. nudicaule* petals supplemented with indole solution. (**A**) HPLC elution profile (460 nm, HPLC 1, method 2) of an extract without the addition of HCl (blue dotted line: 10 min; gray line: 70 min after indole addition) and after adding HCl (dark green line: 10 min; light green line: 70 min after indole addition), indicating the conversion of pelargonidin glucosides into nudicaulins. (**B**) Petal extracts without HCl: the color stays pale orange, indicating a mixture of pelargonidin glucosides and nudicaulins. Petal extracts with HCl: the color changes from bright orange (color of the pelargonidin flavylium cations and nudicaulins in mixture) to bright yellow, the color of nudicaulins.

To mimic the nudicaulin formation in vitro, an aqueous petal extract of the orange *P. nudicaule* cultivar was mixed with indole solution and analyzed by HPLC. Within 70 min, only a marginal conversion of pelargonidin glucosides to nudicaulins occurred. In light of the presumed acidic conditions in the flower, hydrochloric acid was added to another aliquot of the petal extract, decreasing the pH from 5.8 to 1.3. Lowering the pH initiated a structural change of the pelargonidin glucosides to their flavylium cation which was accompanied by an immediate color change from pale to bright orange. Ten min after the addition of indole solution, the HPLC peaks of the pelargonidin glucosides were reduced to about one-third of their original intensity. After 70 min, the peaks of the pelargonidin glucosides had been almost entirely replaced by the peaks of the nudicaulins (Figure 3A). The color change of the extracts illustrates this conversion (Figure 3B). These results demonstrate that the production of nudicaulins in vitro is possible but requires an acidic pH value and a relatively long reaction time.

### 2.2. Synthesis of O-Methylated Nudicaulin Derivatives

According to the “rule of five” for drug-likeness [13], the molecular mass of glucosidic nudicaulins, e.g., nudicaulins I and II (Figure 1, molar mass 871) far exceeds the optimum defined for most pharmacologically active compounds. An in silico drug-likeness prediction using MolCart [14] resulted in positive scores for both nudicaulin glycosides and aglycons. Hence, bioactivity does not necessarily require glycosylation. For these reasons, and for structural simplification, we aimed to synthesize nudicaulin aglycons. However, as shown by hydrolytic experiments, completely deglucosylated nudicaulins are unstable, due to their free hemiacetal structural moiety, and decompose without forming any isolatable compounds [3]. Hence, we describe another structural condition for the nudicaulin derivatives to be synthesized: a small substituent (e.g., methyl) in position 11 of the non-glucosidic structure.

**Scheme 1:**
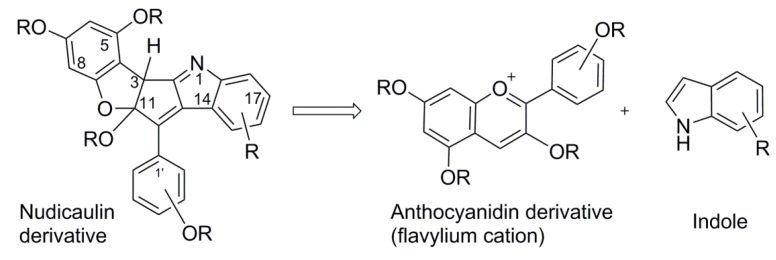
Retrosynthetic analysis of nudicaulin derivatives. R ≠ carbohydrate.

Due to the finding that nudicaulins are biosynthetically formed from pelargonidin glucosides and indole [5], and the presented new results on the spontaneous formation of nudicaulins in vitro, a biomimetic approach following Scheme 1 seemed reasonable. The reductive synthesis from commercially available flavonols was considered the fastest way to prepare the anthocyanidin part. We started with quercetin (**3**), the flavonol analogue of cyanidin, instead of the more expensive kaempferol. Cyanidin possesses an additional hydroxyl group in ring B compared to pelargonidin, the actual precursor of the nudicaulins in *P. nudicaule* (Scheme S1). Precursor-directed biosynthetic (PDB) experiments had already shown that cyanidin can be incorporated into 3′-hydroxynudicaulin. But since the PDB experiments afforded nudicaulin derivatives only in a non-preparative scale [5], this method was unsuitable for the chemical synthesis.

**Scheme 2:**
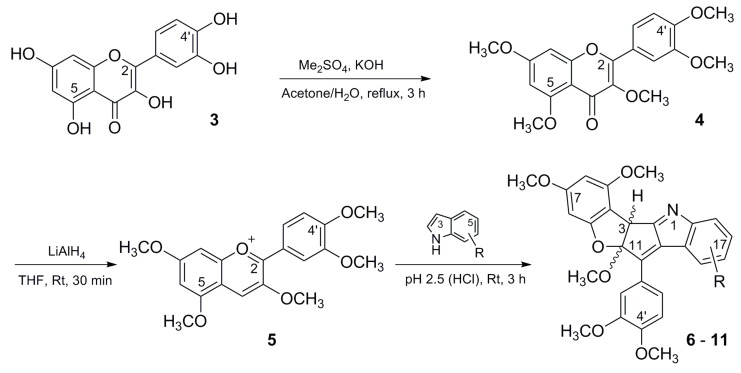
Synthesis of permethylated nudicaulin derivatives including the biomimetic reaction of 3,5,7,3′4′-penta-*O*-methylcyanidin (**5**) with different indoles.

In the first step, quercetin (**3**) was permethylated with Me_2_SO_4_ under alkaline conditions in acetone/water [15]. Reduction of 3,5,7,3′4′-penta-*O*-methylquercetin (**4**) with LiAlH_4_ in THF afforded 3,5,7,3′4′-penta-*O*-methylcyanidin (**5**) [16]. In the final step, compound **5** reacted with indole and yielded a racemic mixture of permethylated nudicaulin diastereomers **6**. The CD spectrum (Figure S43) confirmed the presence of a racemate. The reaction required acidic conditions in order to facilitate formation of a C–C bond between the electrophilic C-2 of **5** and the nucleophilic C-3 of indole (Scheme 2).

Using 3,5,7,3′4′-penta-*O*-methylcyanidin (**5**) as an educt for the polyphenolic part of the nudicaulin molecule and a series of substituted indoles (5-methylindole, 6-methylindole, 7-methylindole, 6-fluoroindole, 5-hydroxyindole) as educts for the nitrogen-containing part, compounds **7** to **11** were obtained. According to the 3′,4′-dihydroxyphenyl ring of starting compound **3**, the skeleton of all synthetic compounds differs from that of the native nudicaulins by an additional hydroxyl group (3′-OH). The structures were confirmed by the characteristic absorption between 460 and 480 nm, mass spectrometry, and 1D and 2D NMR spectroscopic data.

### 2.3. Bioactivity

Compounds **6**, **10** and **11** showed the highest antiproliferative effects against HUVEC (Figure S44) and K-562 (Figure S45) cells and also the strongest cell toxicity against HeLa (Figure S46) cells. The antiproliferative activity (GI_50_) values for compound **6** were 1.3 μmol·L^−1^ against HUVEC and 1.1 μmol·L^−1^ against K-562 cells, the values for compound **10** were 2.2 μmol·L^−1^ and 1.0 μmol·L^−1^ and the values for compound **11** were 2.0 μmol·L^−1^ and 1.0 μmol·L^−1^, respectively. Cytotoxicity varied between 3.4 μmol·L^−1^ (compound **6**) and 5.7 μmol·L^−1^ (compound **10**). Compounds **7**, **8** and **9** exhibited lower antiproliferative activity against HUVEC and K-562 cells, and lower cytotoxicity against HeLa cells. The antiproliferative and cytotoxicity data from all tested compounds are shown in Table S1, with corresponding dose-response curves in Figure S44–46.

No antimicrobial activity against the different bacterial and fungal strains was found for compounds **6**, **7** and **9**–**11** despite the application of high concentrations of 1 mg·mL^−1^. Only compound **8** exhibited marginal activity against *Bacillus subtilis*, *Staphylococcus aureus* (MRSA and non-MRSA variant) as well as *Mycobacterium vaccae*. Detailed results are shown in Table S2.

## 3. Discussion

### 3.1. The Final Step of the Nudicaulin Biosynthesis

Nudicaulins are indole/flavonoid hybrid alkaloids that until now have only been known as natural products from the petals of some Papaveraceous plants such as *P. nudicaule* and *Meconopsis cambrica* [1,17]. Their precursors have been proposed to originate from the phenylpropanoid and the indole biosynthetic pathways [4,5], but the conditions for the final biosynthetic step have remained unknown. Our experiments aimed at clarifying the final step of the nudicaulin formation. We confirmed that pelargonidin glucosides and free indole are the precursors of nudicaulins and demonstrated their biosynthetic fusion by treating intact orange petals with an indole solution. Additionally, the in vitro production of nudicaulins in an aqueous petal extract, which was supplemented with indole, is reported here for the first time. The in vitro reaction was largely pH- and time-dependent. While a slightly acidic pH yielded only a marginal amount of nudicaulins after 70 min, a strongly acidic pH resulted in conversion of approximately 67% of pelargonidins after 10 min and full conversion after 70 min. This result suggests that the pelargonidin glucosides, whose structure also depends on pH value [18], have to be present in their flavylium cation form to react with indole. As the different anthocyanin forms are in equilibrium with each other, a small portion of flavylium cations is also present at pH 6, which explains the small amounts of nudicaulins produced at this pH. Additionally, our investigation of the UV/Vis absorption spectra of *P. nudicaule* petal cell sap revealed that the structure of the nudicaulins might also depend on pH as the spectra varied substantially as pH changed. A spectrum comparable to the one of the living flower was seen only around pH 2. This finding suggests that the vacuoles of *P. nudicaule* petals exhibit a strongly acidic pH value. Even though a spontaneous last step of the nudicaulin biosynthesis seems to be possible under these conditions, subcellular compartmentation studies need to be carried out, because both precursors are present simultaneously during petal development without any nudicaulins being produced [5]. Furthermore, other factors such as transporters for indole, a proton pump to adjust the pH value in the vacuoles, or the stabilization of anthocyanins and possibly nudicaulins by co-pigmentation or stacking may play a role [19,20].

### 3.2. Synthesis of O-Methylated Nudicaulin Derivatives

In view of the activity of other indole alkaloids, the synthesis of nudicaulins appeared a promising way to obtain new compounds with interesting properties, e.g., potential antiproliferative activity. To synthesize nudicaulin derivatives, we chose a biomimetic approach, taking advantage of the spontaneous fusion of anthocyanins with the indole moiety that occurs under strongly acidic conditions. The synthetic route mimics the final step of nudicaulin biosynthesis as it occurs in the petals of the yellow and orange *P. nudicaule* cultivars. Since the glucose moieties of native nudicaulins were considered nonessential for potential bioactivity, we aimed to synthesize aglycon derivatives instead of glucosides. Furthermore, to avoid the instability of the molecules caused by the hemiacetal structure, the hydroxyl group in position 11 had to be protected by methylation, as were the other hydroxyl groups of the cyanidin part of the molecule. Thus, we were successful in the first synthesis of non-glycosylated nudicaulins.

Optimizing the reaction conditions (reaction time and temperature, adjustment of pH, etc.) is assumed to result in improved yield for the final step of the reaction. In general, our work provides easy synthetic access to flavonoidal indole alkaloids.

### 3.3. Bioactivity

The synthetic nudicaulin derivatives **6**–**11** showed high antiproliferative activity against HUVEC and K-562 cells and cytotoxic activity against HeLa cells. Compounds **6**–**11** possessed an activity against K-562 cells similar to doxorubicin, an established chemotherapeutic agent. The antiproliferative activity against HUVEC cells and cytotoxicity against HeLa cells was slightly lower than against K-562 cells (Table S1). However, as the activities varied only little within the same order of magnitude, further investigations are necessary to evaluate the structure–activity relationship of the nudicaulin derivatives in-depth. Our study is suggesting the nudicaulin skeleton as a possible new core structure for further research, aiming at the enhancement of the antiproliferative selectivity.

## 4. Materials and Methods

### 4.1. General Experimental Procedures

Solvents and reagents were purchased from Sigma-Aldrich, Deisenhofen, Germany and used without further purification. All reactions were monitored by thin-layer chromatography (TLC) carried out on 0.25 mm Merck silica gel sheets 60-F_254_ (Merck, Darmstadt, Germany) and visualized by UV light (254 nm).

HPLC 1: The analytical Agilent 1100 HPLC system consisted of a degasser G1322A, binary pump G1312A, autosampler G1313A, and photodiode array detector (PDA) G1315B (Agilent, Waldbronn, Germany) equipped with an EC250/4 Nucleodur C18 HTec column (5 µm; Macherey-Nagel, Düren, Germany). Solvent A was acidified water (0.1% trifluoroacetic acid (TFA) (*v*/*v*)); solvent B, methanol (MeOH); and solvent C, acidified MeOH (0.1% TFA (*v*/*v*)). The column temperature was 25 °C, the flow rate was 1 mL·min^−1^, and the detection wavelengths were 211, 254, 281, 351 and 460 nm. Method 1 included isocratic elution with 30% B/70% A for 20 min followed by a 35-min gradient from 30% to 65% of B with a subsequent washing step (65% to 100% solvent B in 10 min/100% solvent B for 10 min) and equilibration to starting conditions (100% to 30% solvent B in 5 min/30% solvent B for 10 min). Method 2 consisted of a linear binary gradient from 0 to 76% of solvent B in solvent A in 38 min with a subsequent washing step (76% to 100% solvent B in 2 min/ 100% solvent B for 5 min) and equilibration to starting conditions (100% to 0% solvent B in 5 min/0% solvent B for 10 min). Method 3 included a linear binary gradient from 50 to 100% of solvent C in solvent A in 35 min with a subsequent washing step (100% solvent C for 5 min) and equilibration to starting conditions (100% to 50% solvent B in 5 min/50% solvent B for 10 min).

HPLC 2: Preparative HPLC separations were carried out using a Shimadzu Prominence HPLC system (Shimadzu, Duisburg, Germany), controlled by the Shimadzu LCSolution ver. 1.21, and consisting of a DGU-20A5 degasser, LC-20AT gradient pump, SIL-10AP autosampler, CTO-20A column oven, SPD-20A UV detector, FRC-10A fraction collector and CBM-20A system controller. A Nucleodur VP250/10 Nucleodur C18 HTec column (5 µm; Macherey-Nagel, Düren, Germany) was used at 25 °C with a flow rate of 3.5 mL·min^−1^. The binary solvent system consisted of acidified water (0.1% formic acid (FA) (*v*/*v*), solvent A) and acidified MeOH (0.1% FA (*v*/*v*), solvent B). Method 4 included a linear binary gradient from 70–100% B in 15 min with a subsequent washing step (100% solvent B) and fraction collection using intensity and time.

LC 3: Flash chromatography was carried out by a Biotage Isolera Spectra One (Biotage, Uppsala, Sweden) equipped with Biotage Snap-Ultra cartridges (Biotage HP-Sphere, 25 g) and a PDA detector in λ-al detection mode. The binary solvent system consisted of acidified dichloromethane (0.5% TFA (*v*/*v*); solvent A) and acidified methanol (0.5% TFA (*v*/*v*); solvent B). The flow rate was 30 mL·min^−1^. Method 5 consisted of a linear binary gradient from 0–10% B in 34 min, followed by a linear binary gradient from 10–100% in 2 min with a subsequent washing step (100% solvent B) and fraction collection using intensity.

NMR spectra were recorded on a Bruker Avance III 400 MHz spectrometer (operating at 400.13 MHz for ^1^H and 100.61 MHz for ^13^C) equipped with 5 mm BBFO probe (Bruker Biospin, Rheinstetten, Germany) or a Bruker Avance III HD 500 MHz spectrometer (operating at 500.13 MHz for ^1^H and 125.75 MHz for ^13^C) equipped with a 5 mm TCI cryoprobe. Structures were assigned with the aid of ^1^H NMR, ^13^C NMR, APT, ^1^H,^1^H-COSY, HSQC, ^1^H,^13^C-HMBC, and ^1^H,^15^N-HMBC spectra. CDCl_3_ (for compounds **4** and **5**) or MeOH-*d*_4_/1% TFA (for nudicaulin derivatives **6**–**11**) were used as solvents. Chemical shifts were referenced to the residual solvent peaks at δ_H_ 7.26 and δ_C_ 77.0 for CDCl_3_ and δ_H_ 3.31 and δ_C_ 49.15 for MeOH-*d*_4_. Data acquisition and processing were accomplished using TopSpin 3.2 (Bruker Biospin, Rheinstetten, Germany). Standard Bruker pulse sequences as implemented in Bruker TopSpin were used for data acquisition.

For HPLC-HRESIMS measurements, an Agilent Infinity 1260 HPLC was coupled to a Bruker Compact OTOF mass spectrometer (Bruker Daltonics, Bremen, Germany), controlled by the Bruker Compass control suite (version 1.9, Bruker Daltonics, Bremen, Germany) together with Bruker OTOFControl version 4.0 (Bruker Daltonics, Bremen, Germany). The samples were analyzed in the positive ionization mode in the mass range *m*/*z* 50 to 1300 using 30,000 m/Vm resolving power.

UV/Vis absorption spectra were recorded with a Jasco V-550 spectrophotometer (Jasco, Pfungstadt, Germany) between 200 and 700 nm. Blank sample measurements were used for baseline correction.

CD spectra were recorded on a Jasco J-810 spectropolarimeter (Jasco, Pfungstadt, Germany). All compounds were measured in MeOH using a quartz cuvette of 1 mm width. Spectra were recorded between 200 and 600 nm and were accumulated 5 times. Baseline correction was performed on the basis of a MeOH blank sample.

### 4.2. Plant Material

The *Papaver nudicaule* L. plants were raised from seeds of ‘Summer Breeze Yellow’ and ‘Summer Breeze Orange’ (Jelitto Staudensamen, Schwarmstedt, Germany) in soil (clay substratum and TS1 substratum, Klasmann-Deilmann, Geeste, Germany) in the glasshouse facilities of the Max Planck Institute for Chemical Ecology, Jena, Germany. The glasshouse chamber was equipped with Phillips Sun-T Agro 400 Na lights to support an illumination period of 14 h. Temperatures ranged from 19–21 °C in the night and from 21–23 °C during the day with a relative air humidity of 50% to 60%. Plants were irrigated for 6 min every day.

### 4.3. Final Step of Nudicaulin Biosynthesis

In vivo: One petal of the orange *P. nudicaule* cultivar was placed in an aqueous indole solution (1 mg·mL^−1^, Merck, Darmstadt, Germany) for 20 min until its color changed to yellow. Afterwards, the petal and an untreated control petal were extracted with methanol–water mixture 1:1 (20 µL solvent per mg tissue, 30 min ultrasound) and centrifuged (13,200 rpm) for 20 min, after which the supernatant was used for HPLC analysis (HPLC 1, method 1).

In vitro: Petals from the orange cultivar were homogenized in deionized water (10 µL mg^−1^ tissue) by Bertin Minilys Homogenizer (Bertin Technologies, Montigny-le-Bretonneux, France; 120 s, 0.3 g of 1.4 mm ceramic (zirconium oxide) beads, 5000 rpm). After 10 min of ultrasound and 10 min of centrifugation (13,200 rpm), 100 µL of the supernatant was mixed with 10 µL of an aq. indole solution (1 mg·mL^−1^), vortexed and measured by HPLC 1 after 10 min and 70 min (method 2). Additionally, 100 µL of the extract was mixed with 1 µL of hydrochloric acid (37%), intensifying the color. The pH value decreased from 5.8 to 1.2 (measured by a pH-electrode HI1093B, Hanna Instruments, Vöhringen, Germany). Afterwards, 10 µL of aq. indole solution (1 mg·mL^−1^) was added, and after vortexing and 10 or 70 min of reaction time, the samples were measured by HPLC 1 (method 2).

pH dependence of nudicaulin UV/Vis absorption: The cell sap was pressed from the petals of the yellow *P. nudicaule* cultivar and diluted with deionized water (1:2). UV/Vis absorption spectra were recorded in a quartz cell with a thickness of 10 mm. The pH of the sample as well as the blank were adjusted with hydrochloric acid and sodium hydroxide solution (1 mol·L^−1^) using the pH-electrode HI1093B (Hanna Instruments, Vöhringen, Germany). To obtain a spectrum of a petal, the tissue was cut in pieces and its transparency was increased by vacuum infiltration in deionized water [21]. Afterwards, the moistened petal was placed between two slides and measured using the UV/Vis spectrophotometer. The spectra were normalized to a maximal intensity of 1 mAU.

### 4.4. Synthetic Procedures and Analytical Data

#### 4.4.1. 3,5,7,3′4′-Penta-*O*-Methylcyanidin (**5**)

3,5,7,3′4′-Penta-*O*-methylquercetin (**4**) was obtained from quercetin (**3**) by methylation with Me_2_SO_4_ in acetone/water 2:1 according to a reported procedure [22]. Compound **4** was purified by recrystallization from CH_2_Cl_2_/EtOAc (1:6). Reduction of compound **4**, using LiALH_4_ in dry THF under argon atmosphere as described by Kimura et al. [16], afforded 3,5,7,3′4′-penta-*O*-methylcyanidin (**5**). Analytical data of compounds **4** and **5** matched those reported in the literature [16,22].

#### 4.4.2. General Procedure for the Preparation of Nudicaulin Derivatives (Racemic Mixtures)

3,5,7,3′,4′-Penta-*O*-methylcyanidin (1 eq.) was dissolved in a 1:1 solution of MeOH/water at room temperature. The pH of the resulting solution was set to 2.5 by adding 0.1 M HCl aq., followed by a methanolic indole solution (3.0–5.5 eq.). The reaction mixture was stirred for 2.5 h and was stopped by the evaporation of MeOH. The aq. residue was spiked with FA (4 mL) and extracted with DCM (4 × 25 mL). The combined extracts were dried over MgSO_4_, and the solvent was evaporated *in vacuo* until dryness. Purification was done by LC 3, method 5. Final purification was done by preparative HPLC 2, method 4. Evaporation of the solvent by a N_2_ stream and drying *in vacuo* yielded the desired nudicaulin. Purity was verified by HPLC 1, method 3.

#### 4.4.3. 5,7,11,3′,4′-Penta-*O*-Methylnudicaulin (**6**)

3,5,7,3′,4′-Penta-*O*-methylcyanidin (17.8 mg, 0.038 mmol, 1 eq.) dissolved in MeOH/water mixture (1:1, 100 mL) and indole (22.25 mg, 0.19 mmol, 5 eq.) reacted according to the general procedure. The title compound was obtained as a yellow solid (yield: 3.1 mg, 0.007 mmol, 17%). MS: (M + H)_calc_: 472.1755 *m*/*z*, (M + H)_exp_: 472.1772 *m*/*z*, UV/Vis (0.1% TFA-MeOH) *λ*_max_: 210 nm, 472 nm.

#### 4.4.4. 16-Methyl-5,7,11,3′,4′-Penta-*O*-Methylnudicaulin (**7**)

3,5,7,3′,4′-Penta-*O*-methylcyanidin (44.8 mg, 0.095 mmol, 1 eq.) dissolved in MeOH/water mixture (1:1, 350 mL) and 5-methylindole (53.7 mg, 0.41 mmol, 4 eq.) reacted according to the general procedure. The title compound was obtained as a yellow solid (yield: 3 mg, 0.006 mmol, 6.4%). MS: (M + H)_calc_: 486.1911 *m*/*z*, (M + H)_exp_: 486.1913 *m*/*z*, UV/Vis (0.1% TFA-MeOH) *λ*_max_: 209 nm, 469 nm.

#### 4.4.5. 17-Methyl-5,7,11,3′,4′-Penta-*O*-Methylnudicaulin (**8**)

3,5,7,3′,4′-Penta-*O*-methylcyanidin (17.9 mg, 0.038 mmol, 1 eq.) dissolved in MeOH/water mixture (1:1, 100 mL) and 6-methylindole (14.6 mg, 0.11 mmol, 3 eq.) reacted according to the general procedure. The title compound was obtained as a yellow solid (yield: 2.4 mg, 0.005 mmol, 12%). MS: (M + H)_calc_: 486.1911 *m*/*z*, (M + H)_exp_: 486.1929 *m*/*z*, UV/Vis (0.1% TFA-MeOH) *λ*_max_: 206 nm, 473 nm.

#### 4.4.6. 18-Methyl-5,7,11,3′,4′-Penta-*O*-Methylnudicaulin (**9**)

3,5,7,3′,4′-Penta-*O*-methylcyanidin (44.8 mg, 0.095 mmol, 1 eq.) dissolved in MeOH/water mixture (1:1, 350 mL) and 7-methylindole (68.5 mg, 0.52 mmol, 5.5 eq.) reacted according to the general procedure. The title compound was obtained as a yellow solid (yield: 4.5 mg, 0.009 mmol, 10%). MS: (M + H)_calc_: 486.1911 *m*/*z*, (M + H)_exp_: 486.1925 *m*/*z*, UV/Vis (0.1% TFA-MeOH) *λ*_max_: 211 nm, 478 nm.

#### 4.4.7. 17-Fluoro-5,7,11,3′,4′-Penta-*O*-Methylnudicaulin (**10**)

3,5,7,3′,4′-Penta-*O*-methylcyanidin (50 mg, 0.1 mmol; 1 eq.) dissolved in MeOH/water mixture (1:1, 350 mL) and 6-fluoroindole (64.6 mg, 0.5 mmol, 5 eq.) reacted according to the general procedure. The title compound was obtained as a yellow solid (yield: 7.7 mg, 0.016 mmol, 16%). MS: (M + H)_calc_: 490.1660 *m*/*z*, (M + H)_exp_: 490.1668 *m/z*, UV/Vis (0.1% TFA-MeOH) *λ*_max_: 212 nm, 395 nm, 478 nm.

#### 4.4.8. 16-Hydroxy-5,7,11,3′,4′-Penta-*O*-Methylnudicaulin (**11**)

3,5,7,3′,4′-Penta-*O*-methylcyanidin (37.8 mg, 0.08 mmol, 1 eq.) dissolved in MeOH/water mixture (1:1, 350 mL) and 5-hydroxyindole (53.7 mg, 0.40 mmol, 5 eq) reacted according to the general procedure. The title compound was obtained as a yellow solid (yield: 2.6 mg, 0.005 mmol, 7%). MS: (M + H)_calc_: 488.1704 *m*/*z*, (M + H)_exp_: 488.1708 *m*/*z*, UV/Vis (0.1% TFA-MeOH) *λ*_max_: 203 nm, 465 nm.

For ^1^H-NMR data of compounds **6** to **11**, see Table 1, and for ^13^C-NMR data, see Table 2. NMR, MS, and UV/Vis spectra are available in the Supplementary Materials (Figures S1–30, S31–36 and S37–42).

### 4.5. Cell Toxicity

To determine cell toxicity and antiproliferative activity, nudicaulin derivatives **6**–**11** were used in a dimethylsulfoxide solution (conc. 10 mg·mL^−1^). Five replicates were investigated for each assay. Cell toxicity assays were conducted using a HeLa cell line, and antiproliferative activity was assayed using HUVEC and K-562 cell lines. All cell lines are from the Leibniz Institute for Natural Product Research and Infection Biology, Hans Knöll Institute (HKI), Adolf-Reichwein-Straße 23, 07745 Jena, Germany. The test substances were dissolved in dimethylsulfoxide before being diluted in the respective medium of the cells to concentrations between 0.1 and 100 μmol·L^−1^. The adherent cells were harvested at the logarithmic growth phase after soft trypsinization using 0.25% trypsin in phosphate-buffered saline (PBS) containing 0.02% ethylenediaminetetraacetic acid (EDTA). For each experiment, approximately 10,000 cells were seeded with 0.1 mL culture medium per well of the 96-well microplates. HeLa cells were pre-incubated for 48 h prior to the addition of the test compounds, which were carefully diluted on the subconfluent monolayers. Incubation was then conducted in a humidified atmosphere at 37 °C and 5% CO_2_. In the case of K-562 cells, the number of viable cells in every well was determined using the CellTiter-Blue1 assay [23]. The adherent HUVEC and HeLa cells were fixed by glutaraldehyde and stained with a 0.05% solution of methylene blue for 10 min. After gentle washing, the stain was eluted with 0.2 mL of 0.33 n HCl in the wells. The optical densities were measured at 660 nm in a SUNRISE microplate reader (Tecan Trading AG, Männedorf, Switzerland).

### 4.6. Antimicrobial Activity

To determine their antimicrobial activity, nudicaulin derivatives **6**–**11** were used in a methanolic solution (concentration 1 mg·mL^−1^). Antibacterial bioassays were performed in accordance with Krieg et al., 2017 [24] using strains of *Bacillus subtilis*, *Staphylococcus aureus*, *Escherichia coli, Pseudomonas aeruginosa, Enterococcus faecalis* and *Mycobacterium vaccae*. The bacteria were cultivated on standard I nutrient agar (NA I) in Petri dishes at 37 °C. Antifungal bioassays were conducted at 30 °C using strains of *Sporobolomyces salmonicolor* and *Penicillium notatum*, which were cultivated on malt agar (MA), and *Candida albicans,* which was cultivated on yeast morphology agar (YMA). After inoculation, a disc (9 mm in diameter) was removed from the center of the Petri dish and 50 μL of the test solution was added to the cavity. After 18 h of incubation at the respective temperatures, the inhibition areolas were measured. Ciprofloxacin (5 µg·mL^−1^ in deionized water) and amphotericin B (10 µg·mL^−1^ in DMSO/MeOH 1:1) were used as reference compounds against bacterial and fungal strains, respectively.

## Figures and Tables

**Table 1:** ^1^H-NMR data of nudicaulin derivatives **6** to **11** (500 MHz, MeOH-*d*_4_/1% TFA).

Position	6	7	8	9	10	11
	δ, mult., *J* [Hz]	δ, mult., *J* [Hz]	δ, mult., *J* [Hz]	δ, mult., *J* [Hz]	δ, mult., *J* [Hz]	δ, mult., *J* [Hz]
3	5.39, s	5.36, s	5.36, s	5.33, s	5.36, s	5.31, s
6	6.30, d, 1.9	6.30, d, 1.9	6.30, d, 1.8	6.29, d, 1.9	6.30, d, 1.8	6.29, d, 1.9
8	6.25, d, 1.9	6.25, d, 1.9	6.25, d, 1.8	6.25, d, 1.9	6.24, d, 1.8	6.25, d, 1.9
15	8.34, d, 7.8	8.15, brs	8.20, d, 8.2	8.15, dd, 2.1, 6.7	8.29, dd, 8.7, ^4^*J*_H-15/F-17_ = 4.9	7.71, d, 2.3
16	7.60, dd, 7.6, 7.8		7.42, brd, 8.2	7.47, overlap	7.32, ddd, 8.7, 2.3, ^3^*J*_H-16/F-17_ = 8.7	
17	7.68, dd, 7.6, 8.0	7.50, brd, 8.3		7.48, overlap		7.07, dd, 2.3, 8.8
18	7.75, d, 8.0	7.62, d, 8.3	7.55, brs		7.49, dd, 2.3, ^3^*J*_H-18/F-17_ = 8.4	7.53, d, 8.8
2′	7.97, d, 2.0	7.96, d, 2.1	7.95, d, 2.0	7.94, d, 2.0	7.92, d, 2.0	7.90, d, 2.1
5′	7.37, d, 8.7	7.38, d, 8.7	7.35, d, 8.7	7.34, d, 8.6	7.34, d, 8.7	7.34, d, 8.7
6′	8.26, dd, 2.0, 8.7	8.25, dd, 2.1, 8.7	8.23, dd, 2.0, 8.7	8.23, dd, 2.0, 8.6	8.20, dd, 2.0, 8.7	8.18, dd, 2.1, 8.7
5-*O*Me	4.01, s	4.00, s	4.00, s	3.99, s	3.99, s	3.99, s
7-*O*Me	3.76, s	3.76, s	3.76, s	3.75, s	3.75, s	3.76, s
11-*O*Me	3.66, s	3.65, s	3.65, s	3.68, s	3.65, s	3.64, s
3′-*O*Me	3.99, s	3.99, s	3.99, s	3.98, s	3.98, s	3.99, s
4′-*O*Me	4.08, s	4.08, s	4.07, s	4.07, s	4.06, s	4.06, s
16-Me		2.52, s				
17-Me			2.54, s			
18-Me				2.64, s		

**Table 2:** ^13^C-NMR data of nudicaulin derivatives **6** to **11** (125 MHz, MeOH-*d*_4_/1% TFA).

Position	6	7	8	9	10	11
	δ	δ	δ	δ	δ, *J*_13C-19F_ [Hz]	δ
2	178.2	177.7	176.9	178.4	179.8	176.2
3	46.0	45.9	46.0	46.0	46.2	45.8
4	100.6	100.7	100.3	100.9	100.7	100.8
5	159.0	159.0	158.8	158.8	158.7	158.7
6	94.3	94.2	94.2	94.2	94.2	94.2
7	166.0	166.0	166.0	165.9	166.0	166.0
8	90.1	90.1	90.1	90.1	90.1	90.1
9	161.7	161.7	161.7	161.6	161.7	161.6
11	127.8	127.7	127.8	127.8	127.6	127.8
12	169.7	169.2	168.6	169.1	168.8	168.6
13	132.5	132.5	132.5	132.9	132.0	132.9
14	123.1	123.2	120.4	123.1	119.7, d, 2.5	124.5
15	125.8	126.2	125.6	123.3	127.2, d, 10.4	112.3
16	129.0	139.7	129.9	128.9	115.9, d, 24.0	159.5
17	132.1	133.0	143.8	133.7	165.2, d, 251.0	119.2
18	117.7	117.3	118.0	128.4	105.7, d, 27.4	118.4
19	148.9	146.9	149.3	147.8	151.2	141.5
1′	124.5	124.4	124.5	124.4	124.5	124.4
2′	116.4	116.3	116.3	116.2	116.3	116.0
3′	151.3	151.2	151.2	151.2	151.3	151.2
4′	158.9	158.0	158.6	158.7	158.9	158.3
5′	113.3	113.5	113.4	113.4	113.5	113.3
6′	132.0	131.8	131.7	131.7	131.9	131.1
5-OMe	56.6	56.9	56.9	56.9	56.6	56.9
7-OMe	56.5	56.5	56.5	56.4	56.4	56.4
11-OMe	53.7	53.7	53.6	53.7	53.7	53.3
3′-OMe	56.9	56.6	56.6	56.5	56.9	56.6
4′-OMe	57.3	57.3	57.3	57.3	57.3	57.2
16-Me		21.7				
17-Me			22.0			
18-Me				17.1		

## Supplementary Materials

The following are available online. Scheme S1: Formation of nudicaulin derivatives, Figures S1–30: NMR spectra of compounds **6**–**11**, Figures S31–36: HR-MS of compounds **6**–**11**, Figures S37–42: UV/Vis spectra of compounds **6**–**11**, Figure S43: CD spectra of compound **6**, Figure S44–46: Dose-response relationship for antiproliferative effect and cytotoxicity of compounds **6**–**11** on HUVEC, K-562 and HeLa cells, Table S1: Antiproliferative and cytotoxicity data of compounds **6**–**11**, Table S2: Antimicrobial data of compounds **6**–**11**.
