# Supplementary material for: Formation of Nudicaulins In Vivo and In Vitro and the Biomimetic Synthesis and Bioactivity of O-Methylated Nudicaulin Derivatives
Source: Molecules. 2018 Dec 18;23(12):3357. doi: 10.3390/molecules23123357 (PMC6320756; doi:10.3390/molecules23123357)
Supplement: Supplementary file 1 [file molecules-23-03357-s001.pdf]

Formation of Nudicaulins In Vivo and In Vitro and the Biomimetic Synthesis and Bioactivity of *O*-Methylated Nudicaulin Derivatives

Bettina Dudek [1,†], Florian Schnurrer [1,†], Hans-Martin Dahse [2], Christian Paetz [1], Anne-Christin Warskulat [1], Christiane Weigel [2], Kerstin Voigt [2] and Bernd Schneider [1,*]

[1] Max Planck Institute for Chemical Ecology, Hans-Knöll-Str. 8, D-07745 Jena, Germany; bdudek@ice.mpg.de (B.D.); fschnurrer@ice.mpg.de (F.S.); cpaetz@ice.mpg.de (C.P.); anne.warskulat@yahoo.de (A.-C.W.)

[2] Leibniz Institute for Natural Product Research and Infection Biology, Hans Knöll Institute (HKI), Adolf-Reichwein-Straße 23, D-07745 Jena, Germany; hans-martin.dahse@hki-jena.de (H.-M.D.); christiane.weigel@hki-jena.de (C.W.); kerstin.voigt@hki-jena.de (K.V.)

* Correspondence: schneider@ice.mpg.de; Tel.: +49-3641-5549682

† These authors contributed equally to this work.

Supplementary Materials

Table of content

1. Nudicaulin formation **3**
2. NMR spectra **3**
3. HR-MS **19**
4. UV/Vis absorption spectra **21**
5. CD spectrum **24**
6. Cell Toxicity **25**
7. Antimicrobial activity **28**

Table of schemes, figures, and tables

Scheme S1: Formation of nudicaulin derivatives from pelargonidin glucosides in *P. nudicaule* petals. Cyanidin, which possesses one additional hydroxyl group, can also form nudicaulin derivatives. **3**
Figure S1: 5,7,11,3',4'-Penta-*O*-methylnudicaulin (6), ^{1}H-NMR spectrum (500 MHz, 1% TFA - MeOH-*d*$_4$) **4**
Figure S2: 5,7,11,3',4'-Penta-*O*-methylnudicaulin (6), ^{13}C-NMR spectrum (500 MHz, 1% TFA - MeOH-*d*$_4$) **4**
Figure S3: 5,7,11,3',4'-Penta-*O*-methylnudicaulin (6), ^{1}H-^{13}C HSQC spectrum (500 MHz, 1% TFA - MeOH-*d*$_4$) **5**
Figure S4: 5,7,11,3',4'-Penta-*O*-methylnudicaulin (6), ^{1}H-^{13}C HMBC spectrum (500 MHz, 1% TFA - MeOH-*d*$_4$) **5**
Figure S5: 5,7,11,3',4'-Penta-*O*-methylnudicaulin (6), ^{1}H-^{1}H COSY spectrum (500 MHz, 1% TFA- MeOH-*d*$_4$) **6**
Figure S6: 16-Methyl-5,7,11,3',4'-penta-*O*-methylnudicaulin (7), ^{1}H-NMR spectrum (500 MHz, 1% TFA - MeOH-*d*$_4$) **6**
Figure S7: 16-Methyl-5,7,11,3',4'-penta-*O*-methylnudicaulin (7), ^{13}C-NMR spectrum (500 MHz, 1% TFA - MeOH-*d*$_4$) **7**
Figure S8: 16-Methyl-5,7,11,3',4'-penta-*O*-methylnudicaulin (7), ^{1}H-^{13}C HSQC spectrum (500 MHz, 1% TFA - MeOH-*d*$_4$) **7**
Figure S9: 16-Methyl-5,7,11,3',4'-penta-*O*-methylnudicaulin (7), ^{1}H-^{13}C HMBC spectrum (500 MHz, 1% TFA - MeOH-*d*$_4$) **8**
Figure S10: 16-Methyl-5,7,11,3',4'-penta-*O*-methylnudicaulin (7), ^{1}H-^{1}H COSY spectrum (500 MHz, 1% TFA - MeOH-*d*$_4$ **8**
Figure S11: 17-Methyl-5,7,11,3',4'-penta-*O*-methylnudicaulin (8), ^{1}H-NMR spectrum (500 MHz, 1% TFA - MeOH-*d*$_4$) **9**
Figure S12: 17-Methyl-5,7,11,3',4'-penta-*O*-methylnudicaulin (8), ^{13}C-NMR spectrum (500 MHz, 1% TFA - MeOH-*d*$_4$) **9**
Figure S13: 17-Methyl-5,7,11,3',4'-penta-*O*-methylnudicaulin (8), ^{1}H-^{13}C HSQC spectrum (500 MHz, 1% TFA - MeOH-*d*$_4$)10

Figure S14: 17-Methyl-5,7,11,3′,4′-penta-*O*-methylnudicaulin (8), ^{1}H-^{13}C HMBC spectrum (500 MHz, 1% TFA - MeOH-*d*$_4$) 10
Figure S15: 17-Methyl-5,7,11,3′,4′-penta-*O*-methylnudicaulin (8), ^{1}H-^{1}H COSY spectrum (500 MHz, 1% TFA - MeOH-*d*$_4$) 11
Figure S16: 18-Methyl-5,7,11,3′,4′-penta-*O*-methylnudicaulin (9), ^{1}H-NMR spectrum (500 MHz, 1% TFA - MeOH-*d*$_4$) 11
Figure S17: 18-Methyl-5,7,11,3′,4′-penta-*O*-methylnudicaulin (9), ^{13}C-NMR spectrum (500 MHz, 1% TFA - MeOH-*d*$_4$) 12
Figure S18: 18-Methyl-5,7,11,3′,4′-penta-*O*-methylnudicaulin (9), ^{1}H-^{13}C HSQC spectrum (500 MHz, 1% TFA - MeOH-*d*$_4$) 12
Figure S19: 18-Methyl-5,7,11,3′,4′-penta-*O*-methylnudicaulin (9), ^{1}H-^{13}C HMBC spectrum (500 MHz, 1% TFA - MeOH-*d*$_4$) 13
Figure S20: 18-Methyl-5,7,11,3′,4′-penta-*O*-methylnudicaulin (9), ^{1}H-^{1}H COSY spectrum (500 MHz, 1% TFA - MeOH-*d*$_4$) 13
Figure S21: 17-Fluoro-5,7,11,3′,4′-penta-*O*-methylnudicaulin (10), ^{1}H-NMR spectrum (500 MHz, 1% TFA - MeOH-*d*$_4$) 14
Figure S22: 17-Fluoro-5,7,11,3′,4′-penta-*O*-methylnudicaulin (8), ^{13}C-NMR spectrum (500 MHz, 1% TFA - MeOH-*d*$_4$) 14
Figure S23: 17-Fluoro-5,7,11,3′,4′-penta-*O*-methylnudicaulin (8), ^{1}H-^{13}C HSQC spectrum (500 MHz, 1% TFA - MeOH-*d*$_4$) 15
Figure S24: 17-Fluoro-5,7,11,3′,4′-penta-*O*-methylnudicaulin (8), ^{1}H-^{13}C HMBC spectrum (500 MHz, 1% TFA - MeOH-*d*$_4$) 15
Figure S25: 17-Fluoro-5,7,11,3′,4′-penta-*O*-methylnudicaulin (10), ^{1}H-^{1}H COSY spectrum (500 MHz, 1% TFA - MeOH-*d*$_4$) 16
Figure S26: 17-Hydroxy-5,7,11,3′,4′-penta-*O*-methylnudicaulin (11), ^{1}H-NMR spectrum (500 MHz, 1% TFA - MeOH-*d*$_4$) 16
Figure S27: 17-Hydroxy-5,7,11,3′,4′-penta-*O*-methylnudicaulin (11), ^{13}C-NMR spectrum (500 MHz, 1% TFA - MeOH-*d*$_4$) 17
Figure S28: 17-Hydroxy-5,7,11,3′,4′-penta-*O*-methylnudicaulin (11), ^{1}H-^{13}C HSQC spectrum (500 MHz, 1% TFA - MeOH-*d*$_4$) 17
Figure S29: 17-Hydroxy-5,7,11,3′,4′-penta-*O*-methylnudicaulin (11), ^{1}H-^{13}C HMBC spectrum (500 MHz, 1% TFA - MeOH-*d*$_4$) 18
Figure S30: 17-Hydroxy-5,7,11,3′,4′-penta-*O*-methylnudicaulin (10), ^{1}H-^{1}H COSY spectrum (500 MHz, 1% - MeOH-*d*$_4$) 18
Figure S31: 5,7,11,3′,4′-Penta-*O*-methylnudicaulin (6), mass spectrum 19
Figure S32: 16-Methyl-5,7,11,3′,4′-penta-*O*-methylnudicaulin (7), mass spectrum 19
Figure S33: 17-Methyl-5,7,11,3′,4′-penta-*O*-methylnudicaulin (8), mass spectrum 19
Figure S34: 18-Methyl-5,7,11,3′,4′-penta-*O*-methylnudicaulin (9), mass spectrum 20
Figure S35: 17-Fluoro-5,7,11,3′,4′-penta-*O*-methylnudicaulin (10), mass spectrum 20
Figure S36: 17-Hydroxy-5,7,11,3′,4′-penta-*O*-methylnudicaulin (11), mass spectrum 20
Figure S37: 5,7,11,3′,4′-Penta-*O*-methylnudicaulin (6), UV/Vis spectrum (0.1 % TFA-MeOH) 21
Figure S38: 16-Methyl-5,7,11,3′,4′-penta-*O*-methylnudicaulin (7), UV/Vis spectrum (0.1 % TFA-MeOH) 21
Figure S39: 17-Methyl-5,7,11,3′,4′-penta-*O*-methylnudicaulin (8), UV/Vis spectrum (0.1 % TFA-MeOH) 22
Figure S40: 18-Methyl-5,7,11,3′,4′-penta-*O*-methylnudicaulin (9), UV/Vis spectrum (0.1 % TFA-MeOH) 22
Figure S41: 17-Fluoro-5,7,11,3′,4′-penta-*O*-methylnudicaulin (10), UV/Vis spectrum (0.1 % TFA-MeOH) 23
Figure S42: 17-Hydroxy-5,7,11,3′,4′-penta-*O*-methylnudicaulin (11), UV/Vis spectrum (0.1 % TFA-MeOH) 23
Figure S43: 5,7,11,3′,4′-Penta-*O*-methylnudicaulin (6), CD spectrum (concentration 0.78 mg ml^{-1} (1.66 mM in MeOH), cuvette width 1 mm) 24
Figure S44: Dose–response relationship for antiproliferative effect on HUVEC cells (72 h), Representative curves are given. 26
Figure S45: Dose–response relationship values for antiproliferative effect on K-562 cells (72 h), Representative curves are given. 26
Figure S46: Dose–response relationship values for cytotoxicity on HeLa cells (72 h), Representative curves are given. 27

Table S1: Antiproliferative and cytotoxicity data of compounds 6-11 25
Table S2: Antimicrobial data of compounds 6-11 28

1. Nudicaulin formation

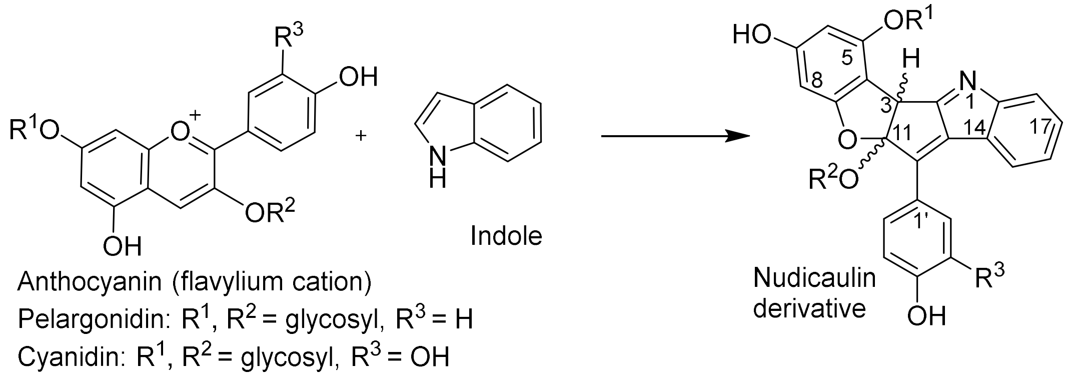

Scheme S1: Formation of nudicaulin derivatives from pelargonidin glucosides in *P. nudicaule* petals. Cyanidin, which possesses one additional hydroxyl group, can also form nudicaulin derivatives.

2. NMR spectra

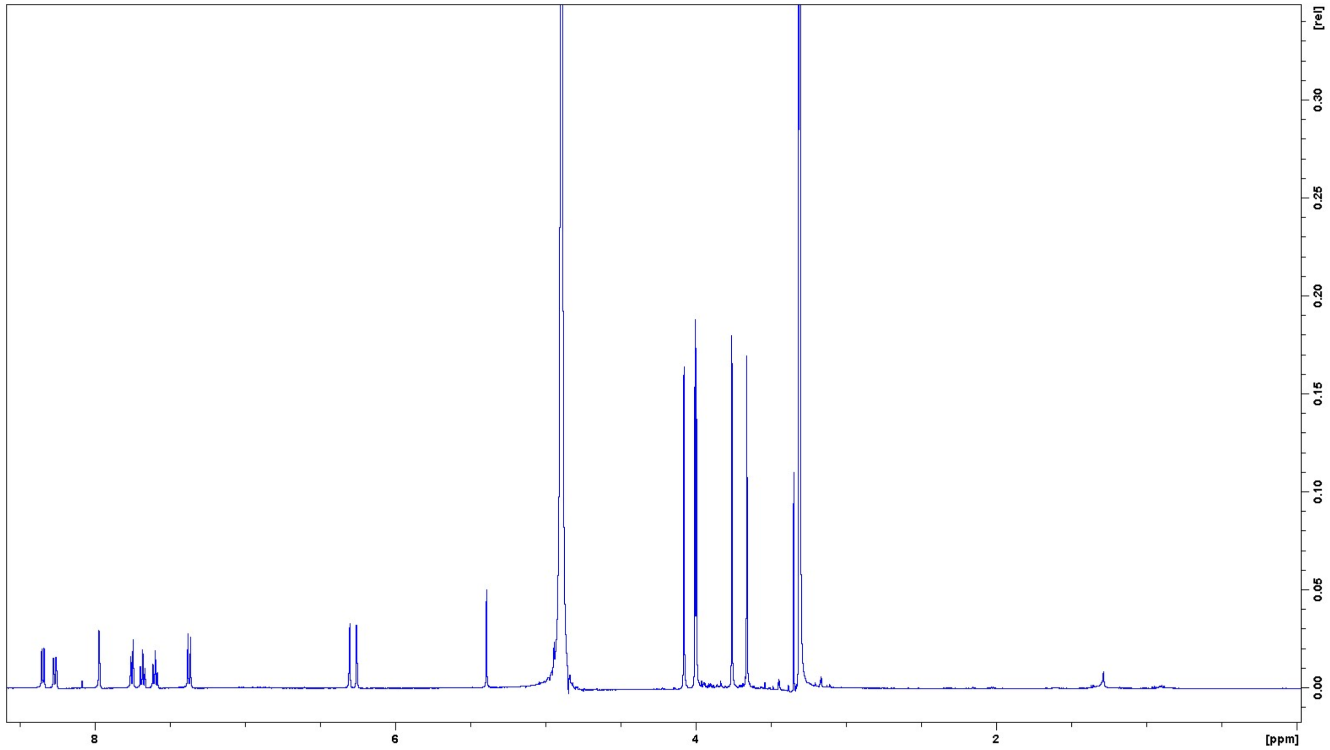

Figure S1: 5,7,11,3′,4′-Penta-*O*-methylnudicaulin (**6**), ^{1}H-NMR spectrum (500 MHz, 1% TFA - MeOH-d_4)

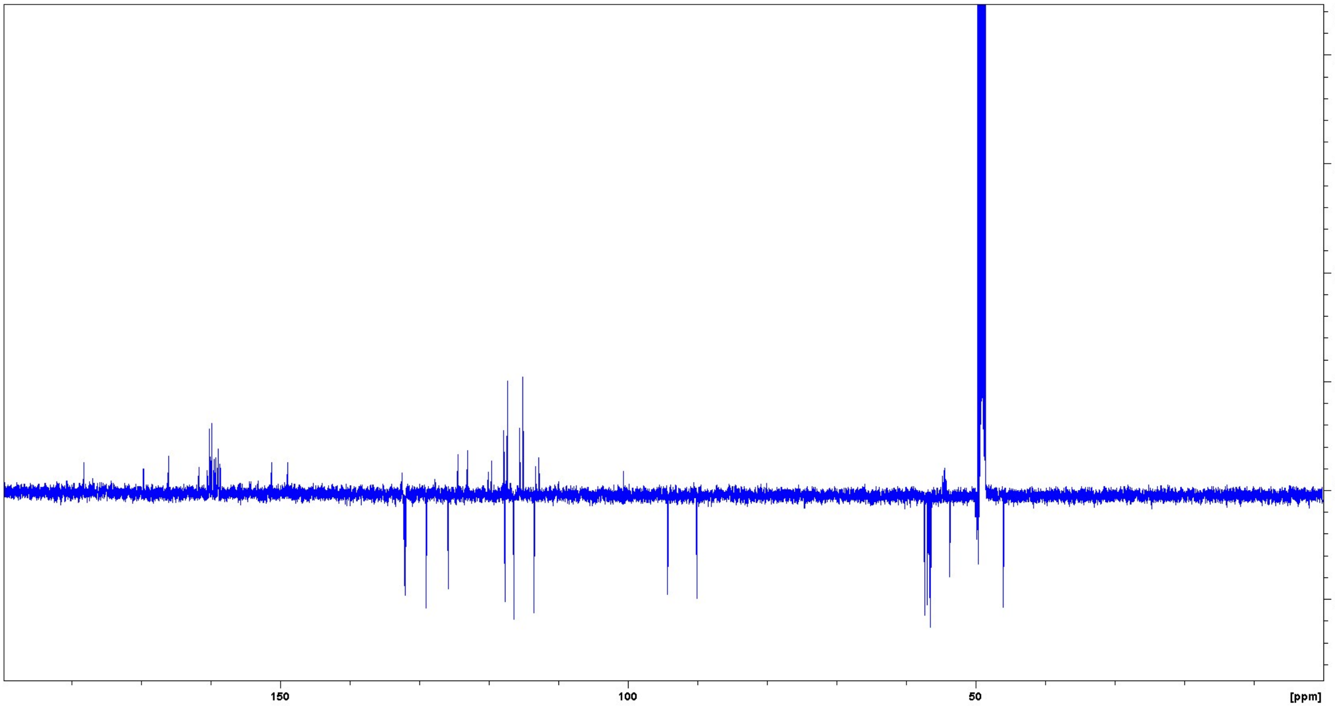

Figure S2: 5,7,11,3′,4′-Penta-*O*-methylnudicaulin (**6**), ^{13}C-NMR spectrum (500 MHz, 1% TFA - MeOH-d_4)

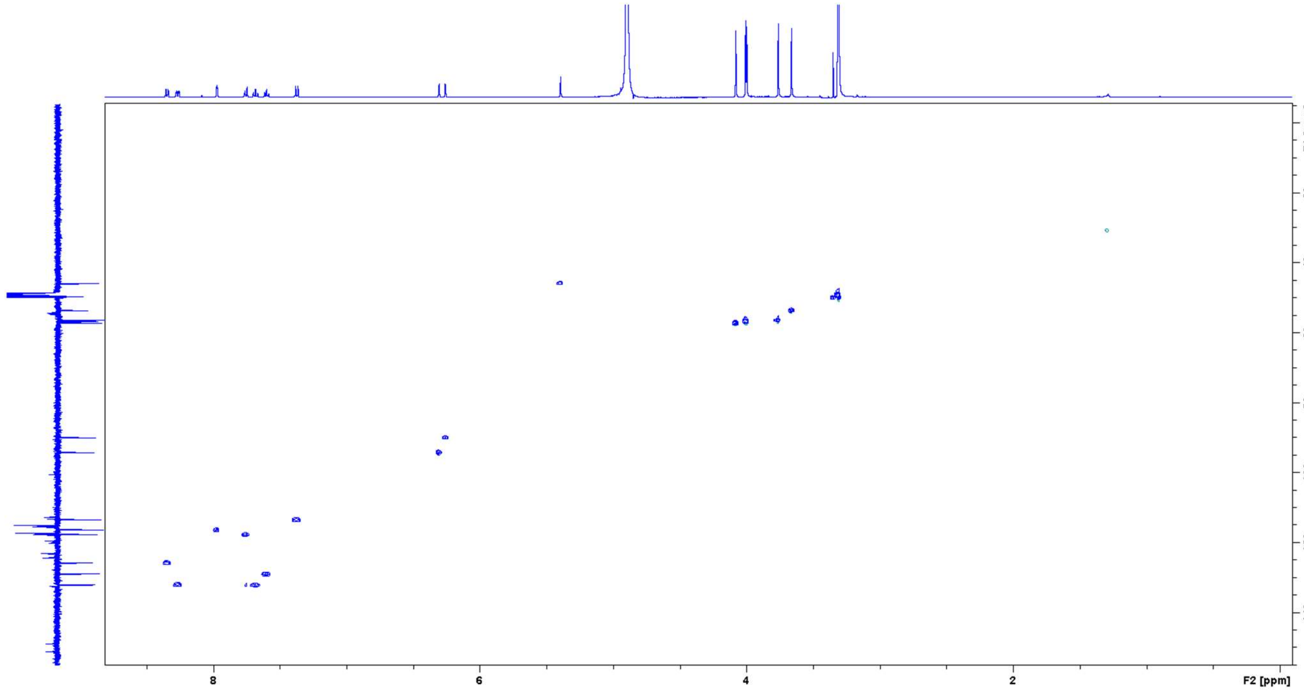

Figure S3: 5,7,11,3′,4′-Penta-*O*-methylnudicaulin (**6**), ^{1}H-^{13}C HSQC spectrum (500 MHz, 1% TFA - MeOH-d_4)

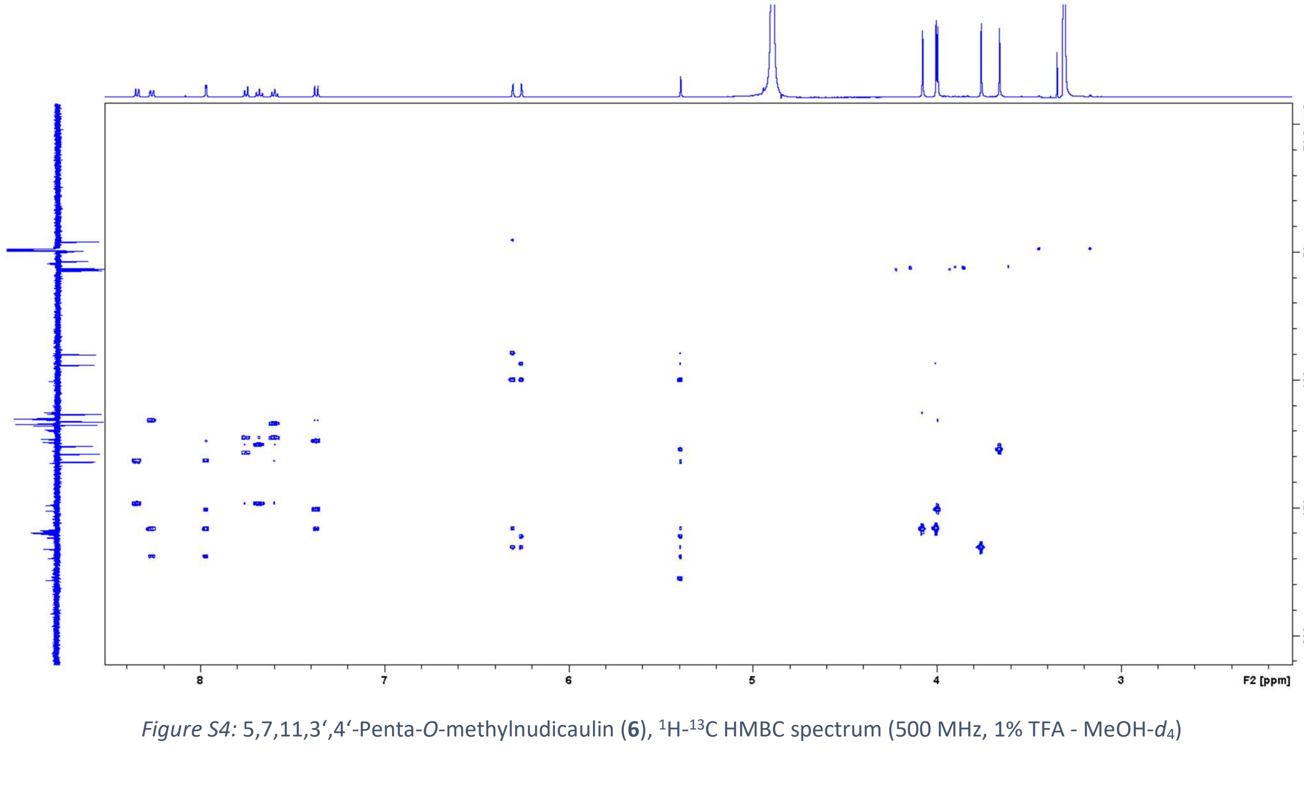

Figure S4: 5,7,11,3′,4′-Penta-*O*-methylnudicaulin (**6**), ^{1}H-^{13}C HMBC spectrum (500 MHz, 1% TFA - MeOH-d_4)

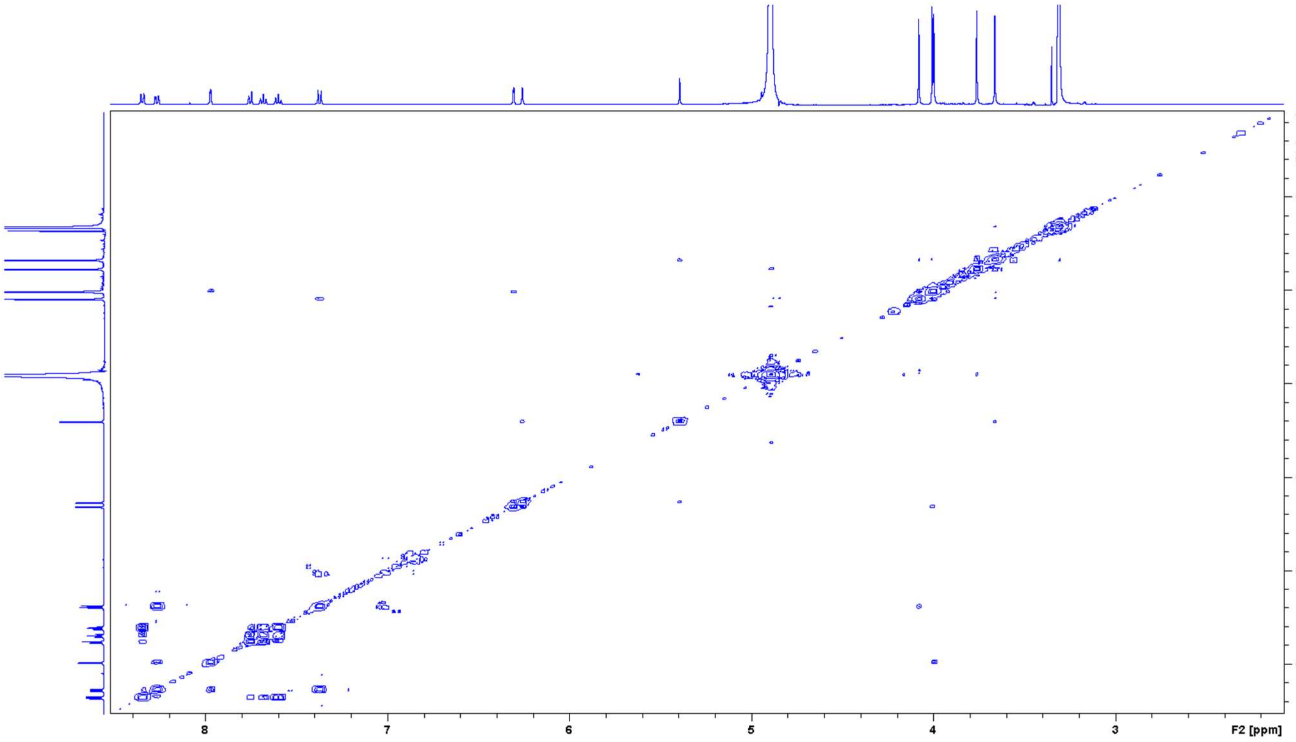

Figure S5: 5,7,11,3′,4′-Penta-*O*-methylnudicaulin (**6**), ^{1}H-^{1}H COSY spectrum (500 MHz, 1% TFA- MeOH-d_4)

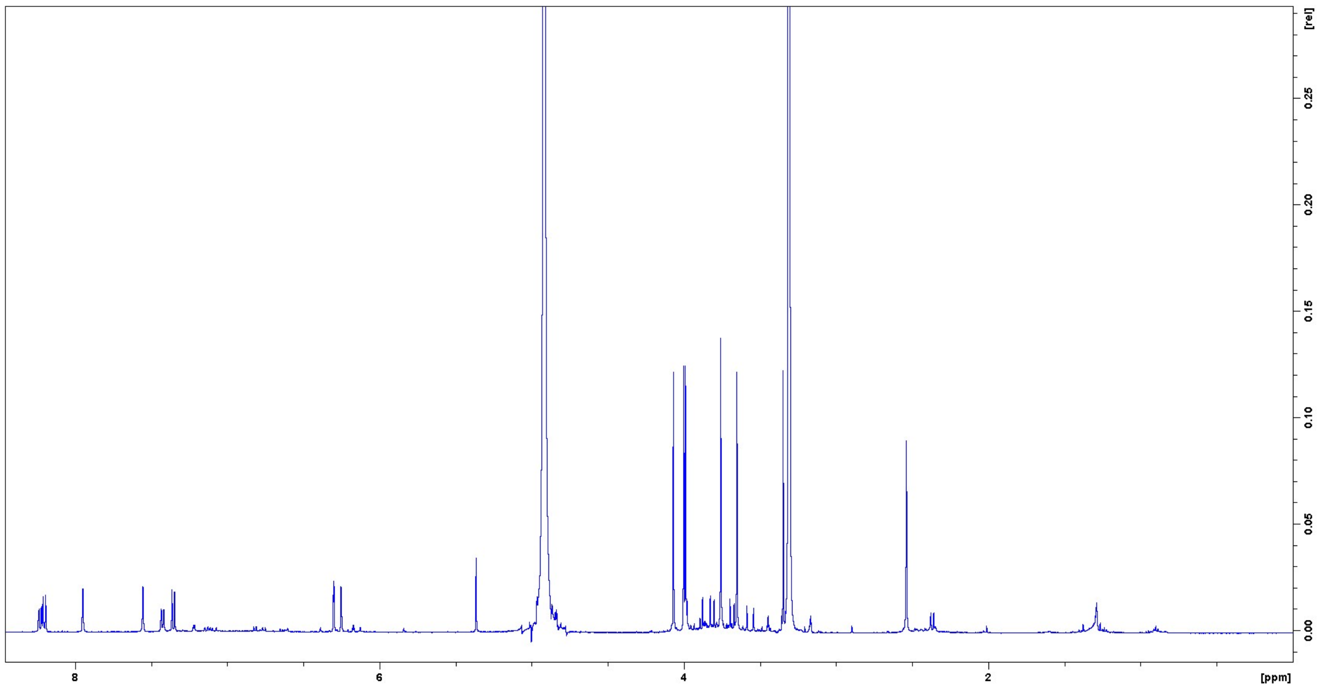

Figure S6: 16-Methyl-5,7,11,3′,4′-penta-*O*-methylnudicaulin (**7**), ^{1}H-NMR spectrum (500 MHz, 1% TFA - MeOH-d_4)

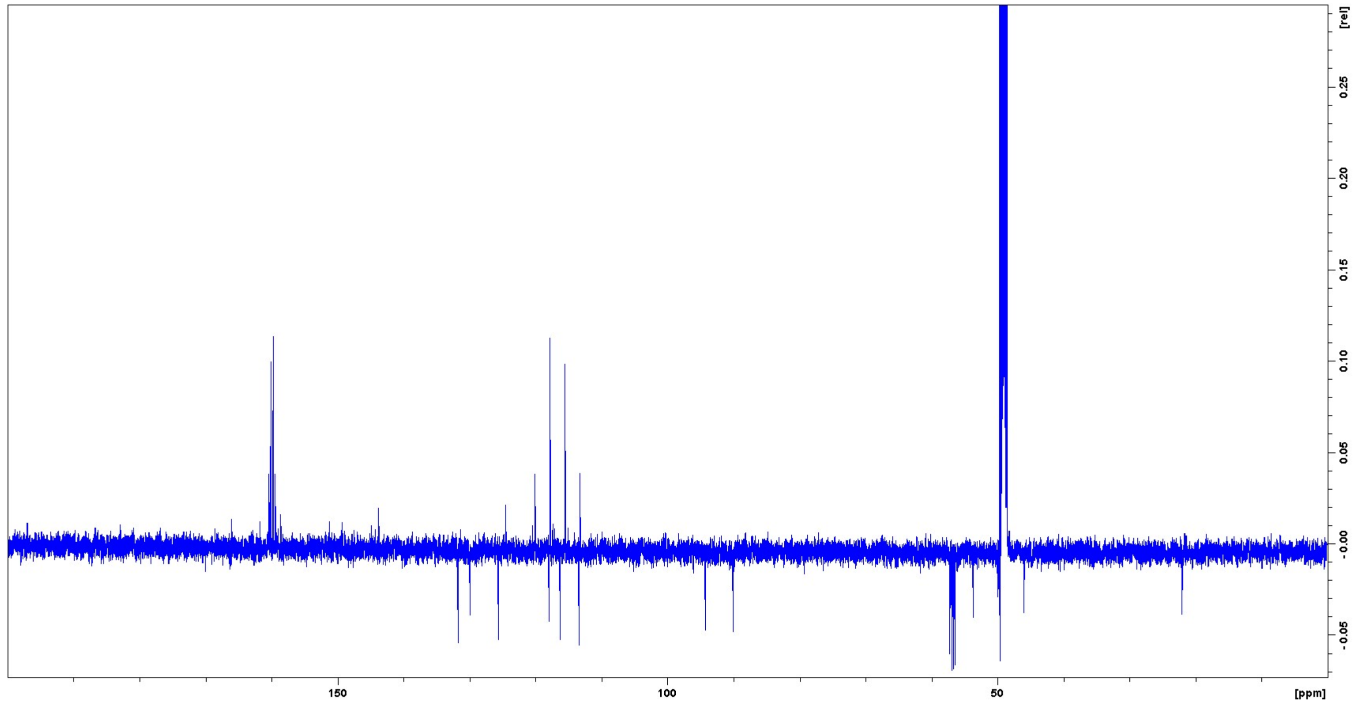

Figure S7: 16-Methyl-5,7,11,3‘,4‘-penta-*O*-methylnudicaulin (**7**), ^{13}C-NMR spectrum (500 MHz, 1% TFA - MeOH-d_4)

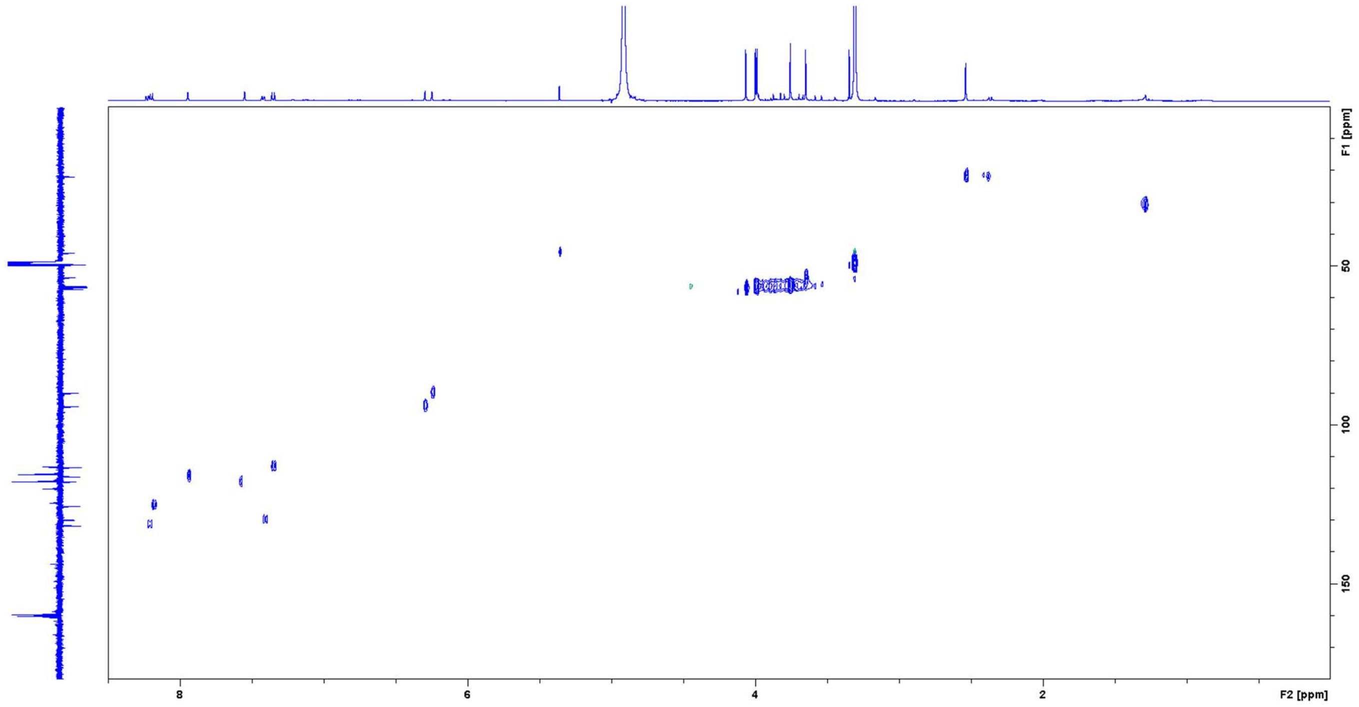

Figure S8: 16-Methyl-5,7,11,3‘,4‘-penta-*O*-methylnudicaulin (**7**), ^{1}H-^{13}C HSQC spectrum (500 MHz, 1% TFA - MeOH-d_4)

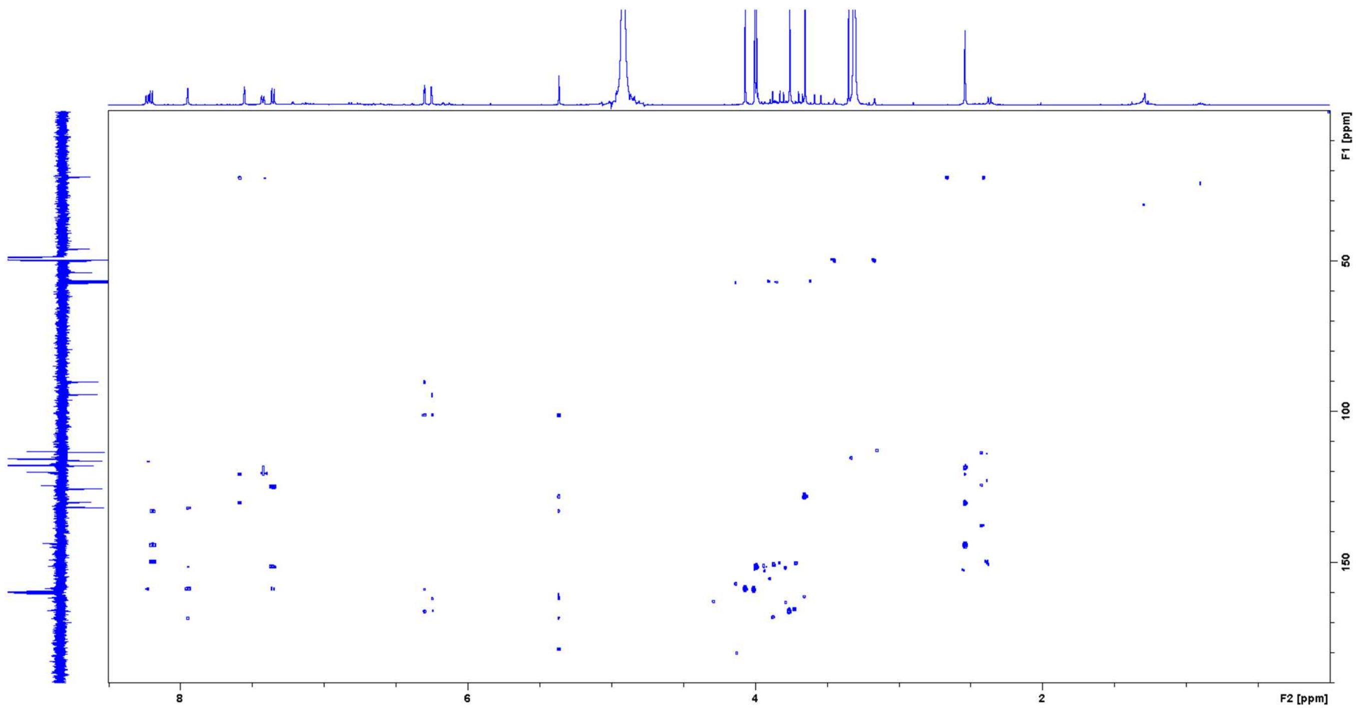

Figure S9: 16-Methyl-5,7,11,3‘,4‘-penta-*O*-methylnudicaulin (**7**), ^{1}H-^{13}C HMBC spectrum (500 MHz, 1% TFA - MeOH-d_4)

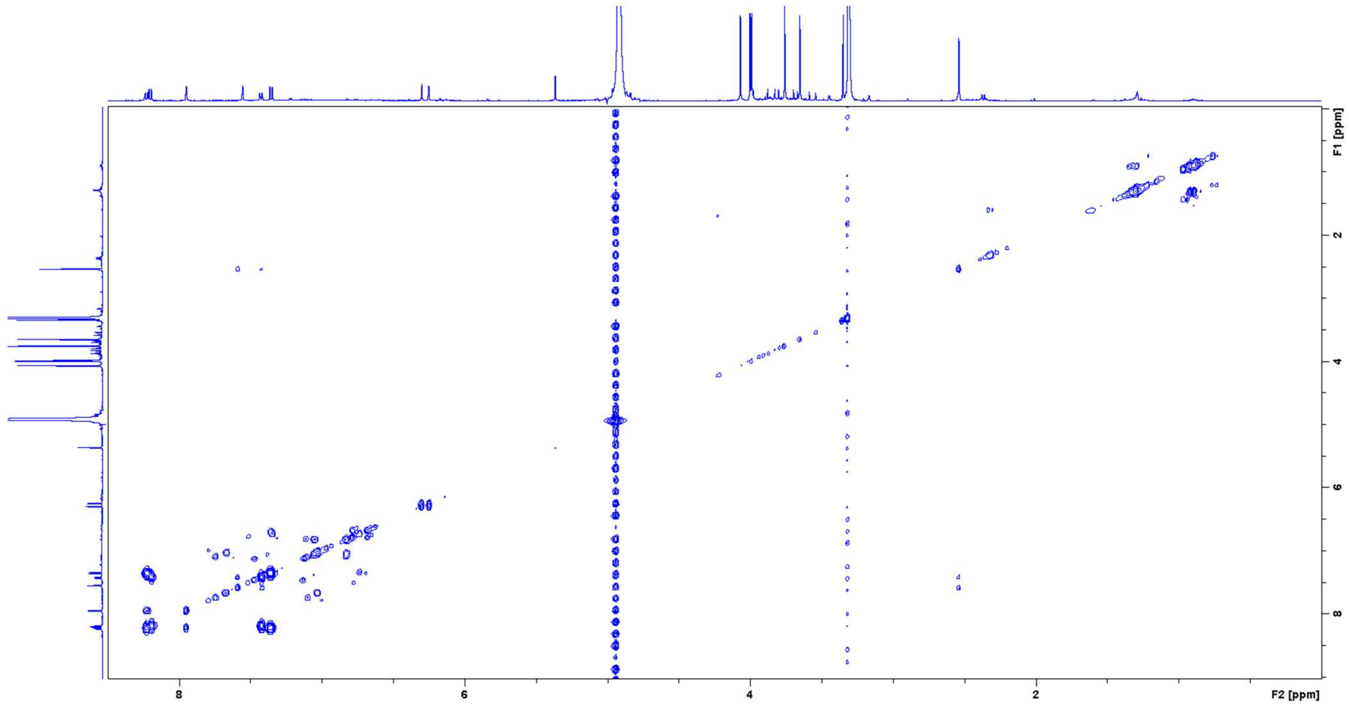

Figure S10: 16-Methyl-5,7,11,3‘,4‘-penta-*O*-methylnudicaulin (**7**), ^{1}H-^{1}H COSY spectrum (500 MHz, 1% TFA - MeOH-d_4

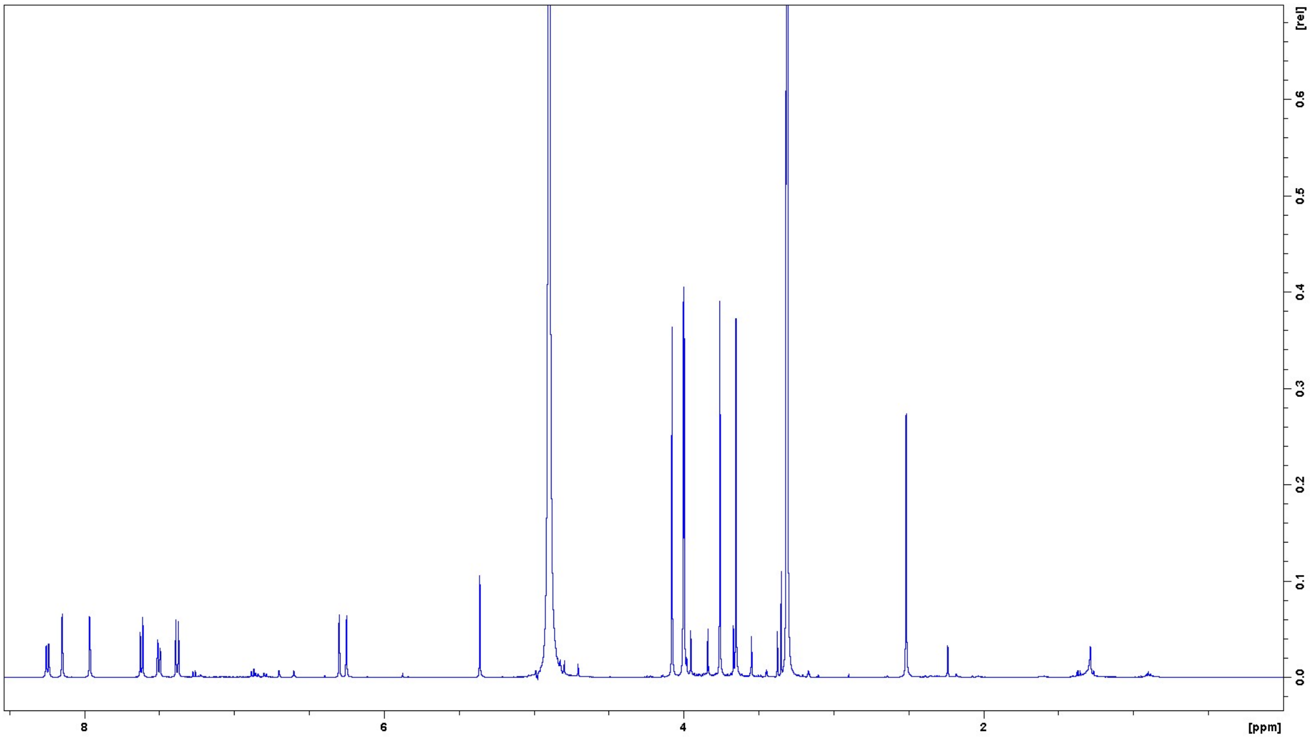

Figure S11: 17-Methyl-5,7,11,3‘,4‘-penta-*O*-methylnudicaulin (**8**), ^{1}H-NMR spectrum (500 MHz, 1% TFA - MeOH-d_4)

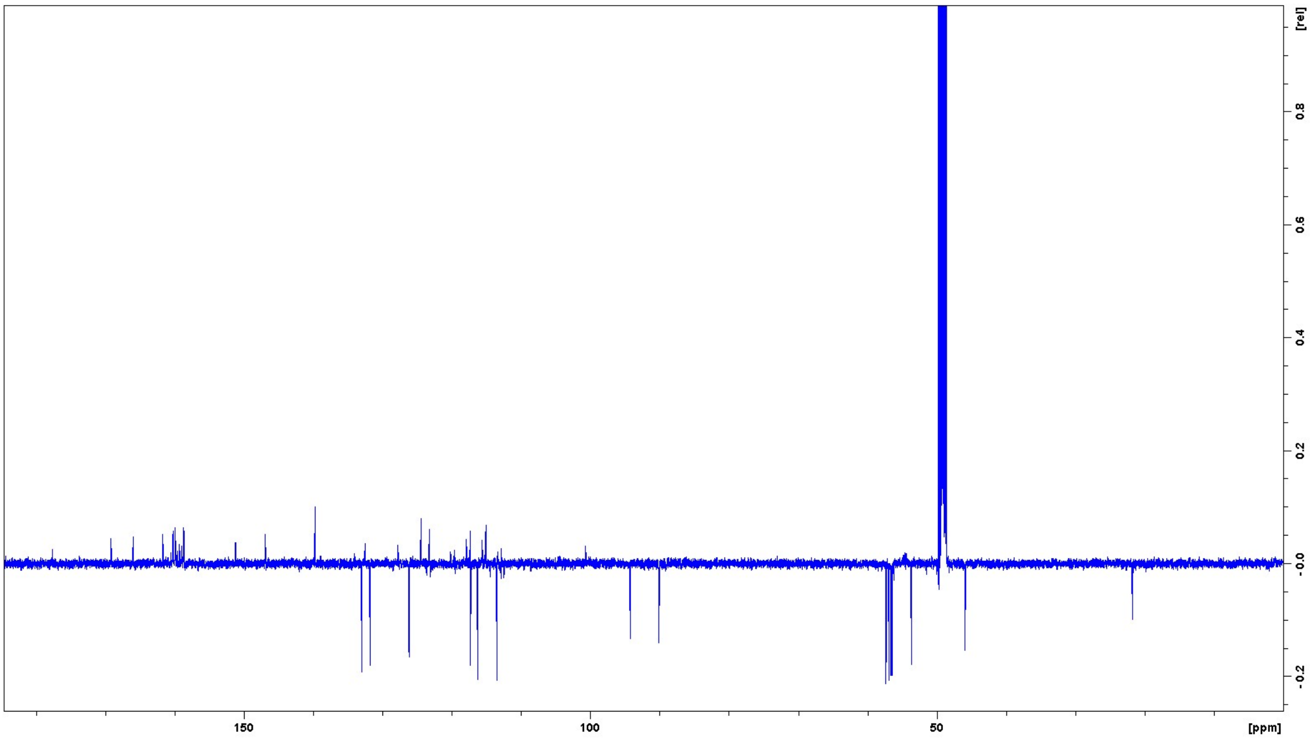

Figure S12: 17-Methyl-5,7,11,3‘,4‘-penta-*O*-methylnudicaulin (**8**), ^{13}C-NMR spectrum (500 MHz, 1% TFA - MeOH-d_4)

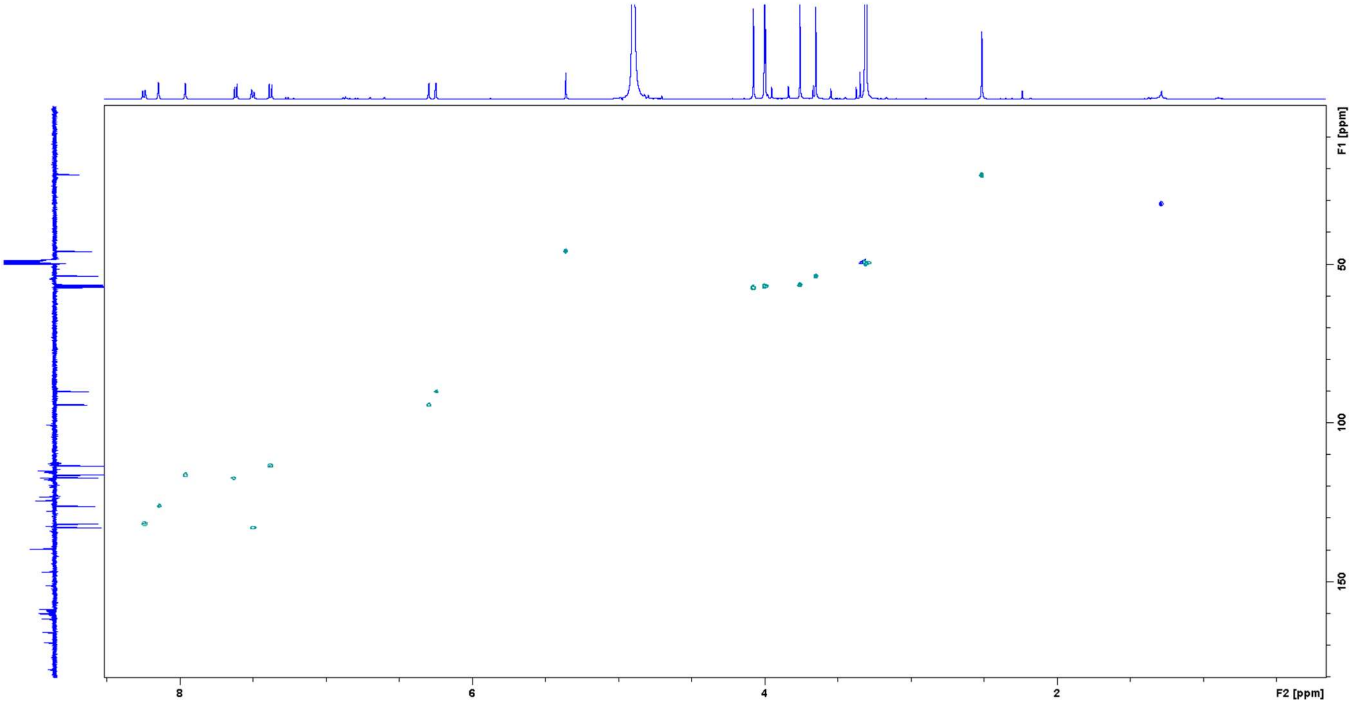

Figure S13: 17-Methyl-5,7,11,3‘,4‘-penta-*O*-methylnudicaulin (**8**), ^{1}H-^{13}C HSQC spectrum (500 MHz, 1% TFA - MeOH-d_4)

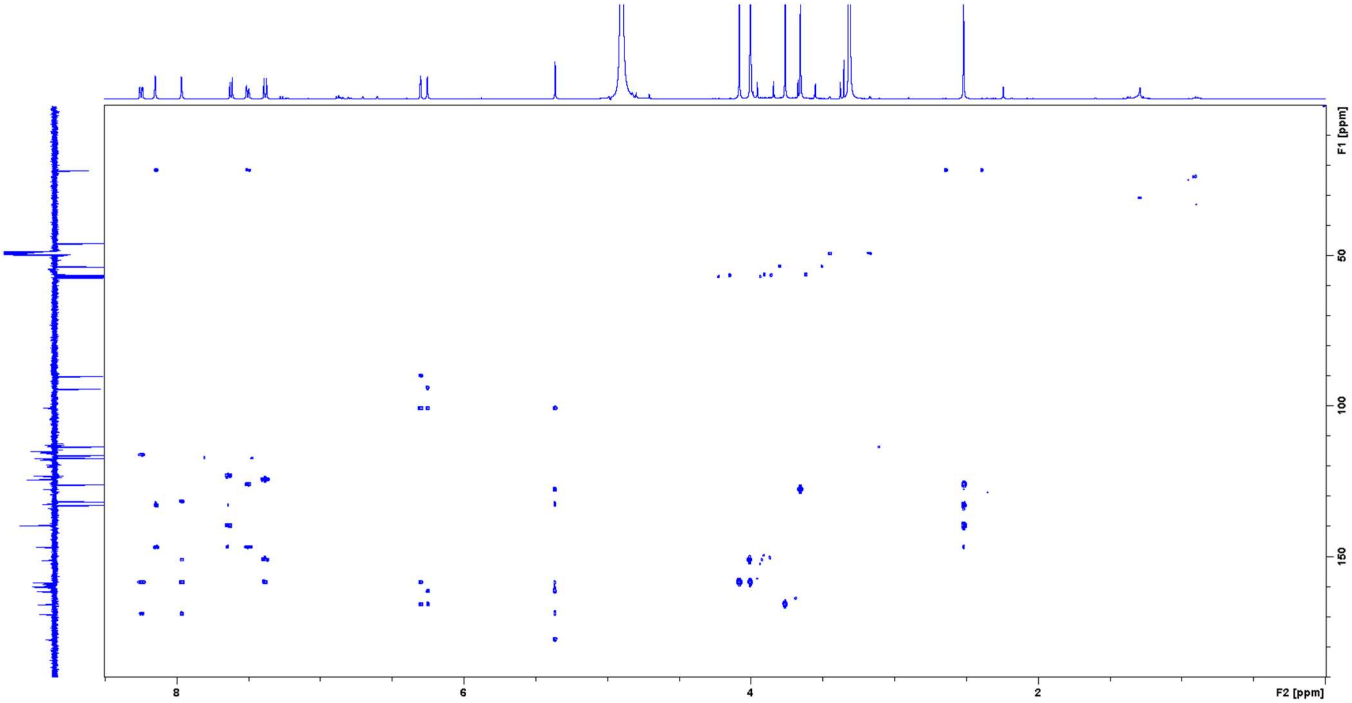

Figure S14: 17-Methyl-5,7,11,3‘,4‘-penta-*O*-methylnudicaulin (**8**), ^{1}H-^{13}C HMBC spectrum (500 MHz, 1% TFA - MeOH-d_4)

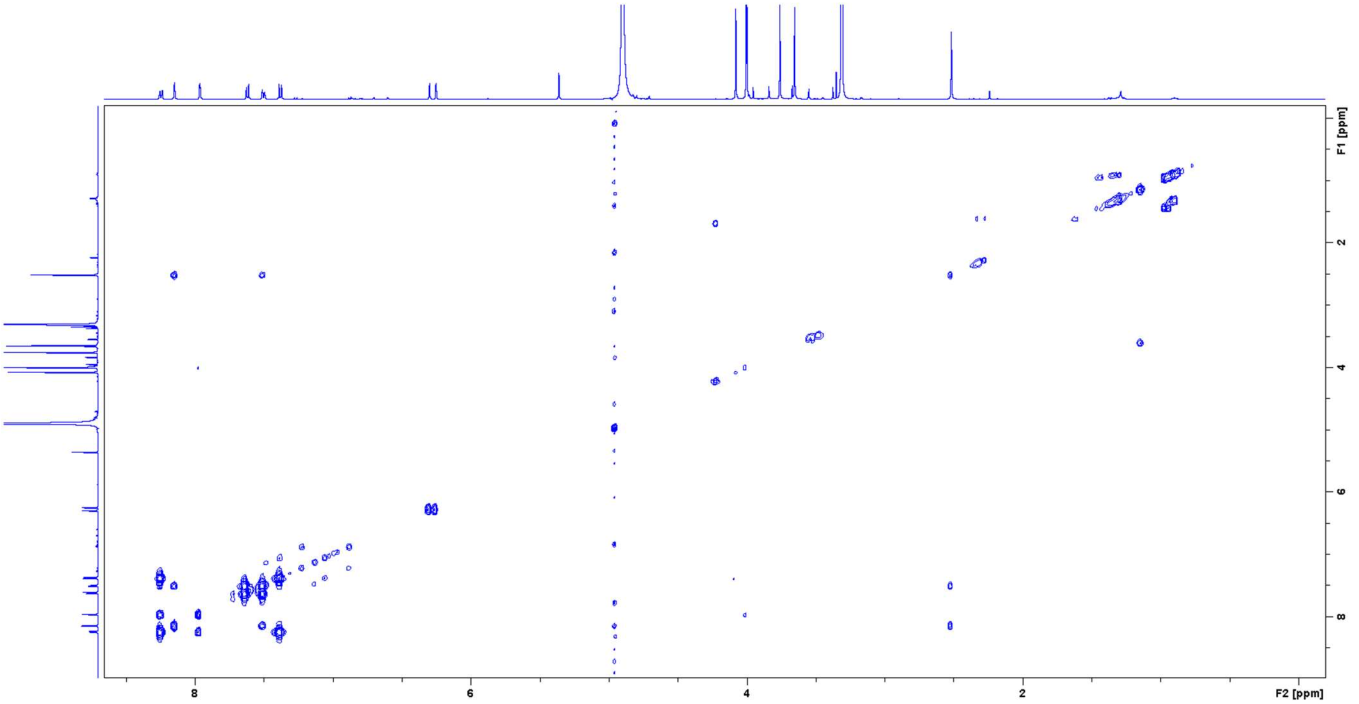

Figure S15: 17-Methyl-5,7,11,3‘,4‘-penta-*O*-methylnudicaulin (**8**), ^{1}H-^{1}H COSY spectrum (500 MHz, 1% TFA - MeOH-d_4)

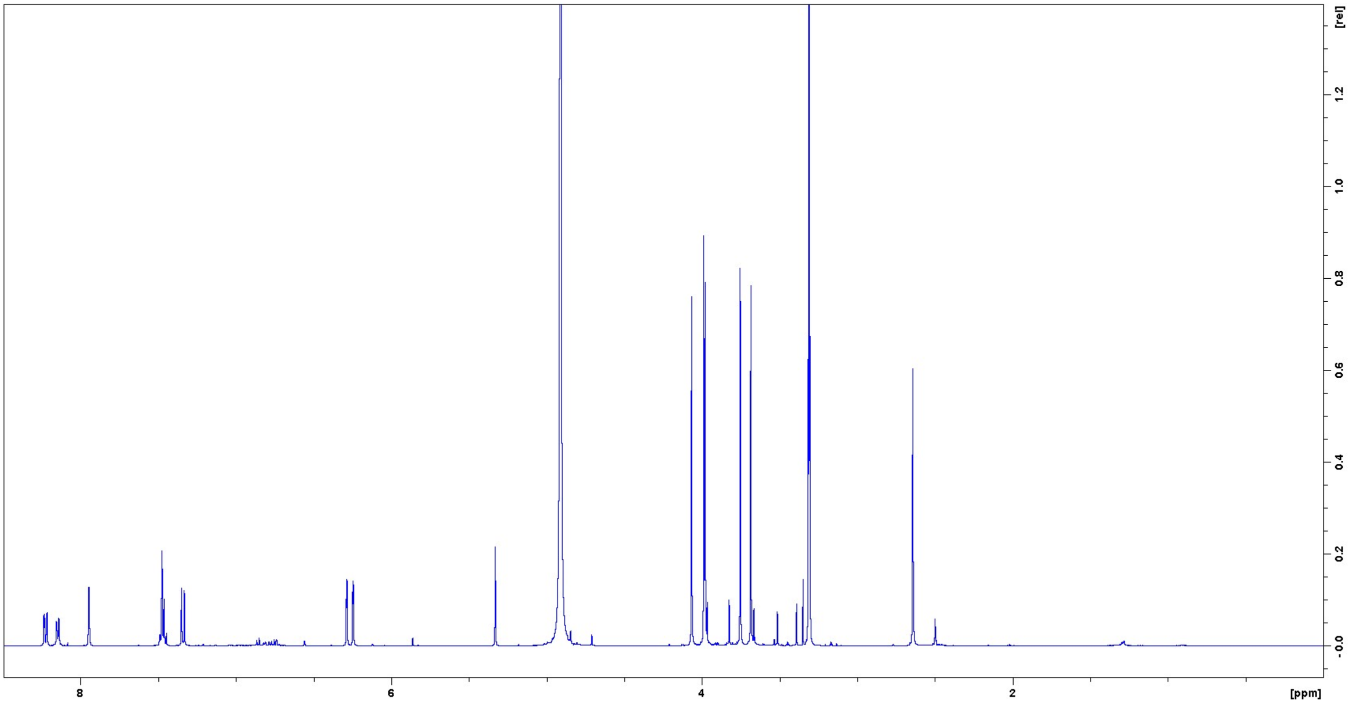

Figure S16: 18-Methyl-5,7,11,3‘,4‘-penta-*O*-methylnudicaulin (**9**), ^{1}H-NMR spectrum (500 MHz, 1% TFA - MeOH-d_4)

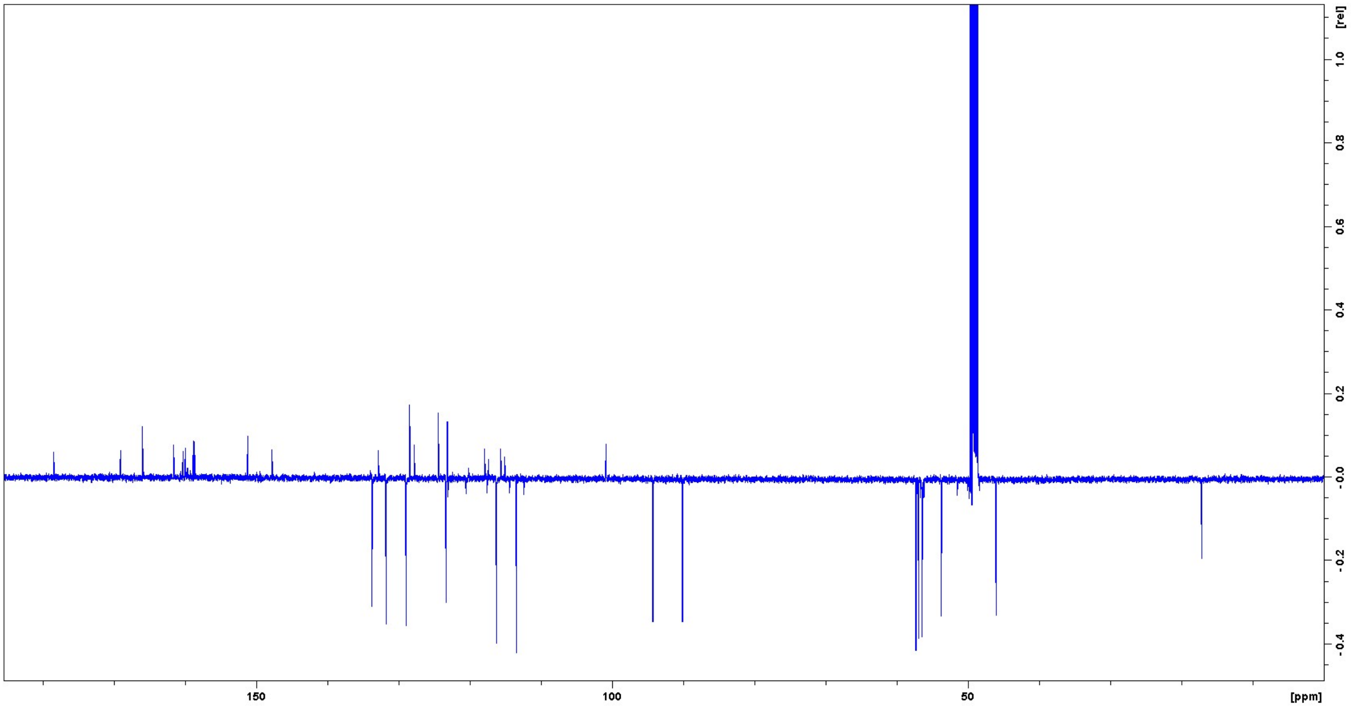

Figure S17: 18-Methyl-5,7,11,3',4'-penta-*O*-methylnudicaulin (**9**), ^{13}C-NMR spectrum (500 MHz, 1% TFA - MeOH-d_4)

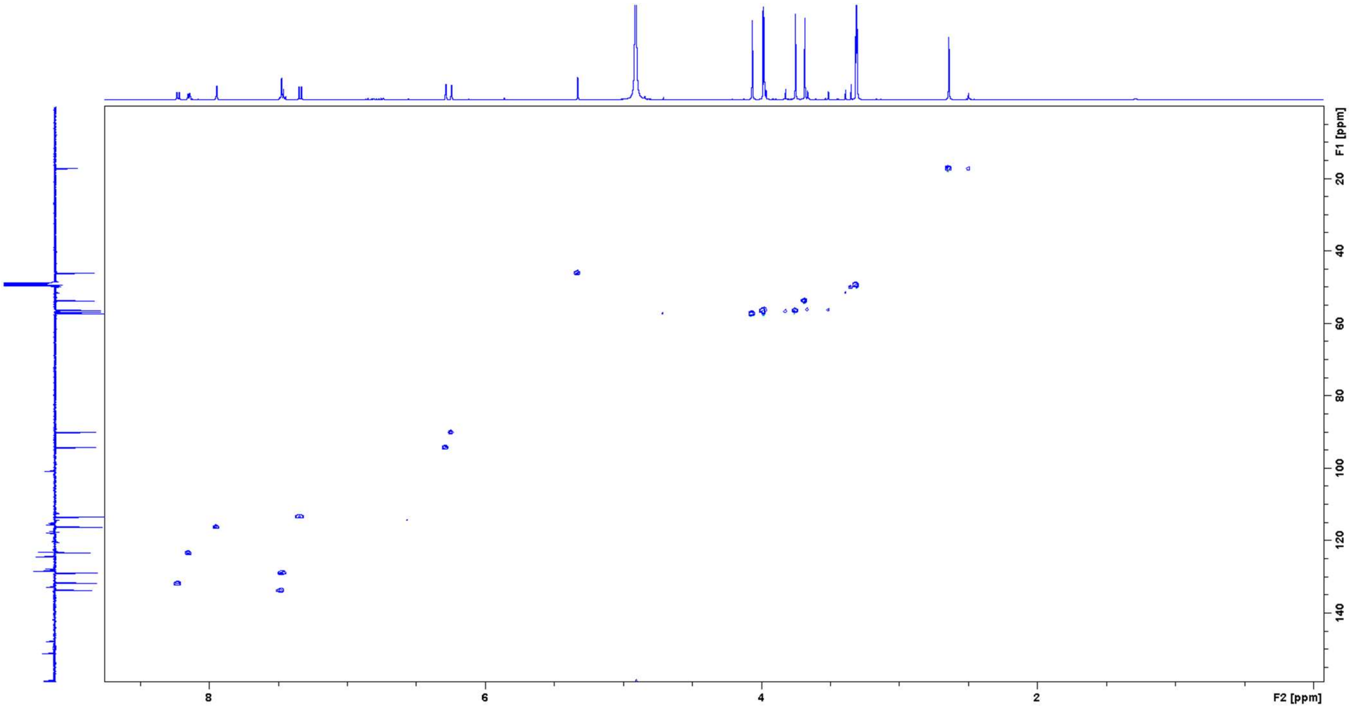

Figure S18: 18-Methyl-5,7,11,3',4'-penta-*O*-methylnudicaulin (**9**), ^{1}H-^{13}C HSQC spectrum (500 MHz, 1% TFA - MeOH-d_4)

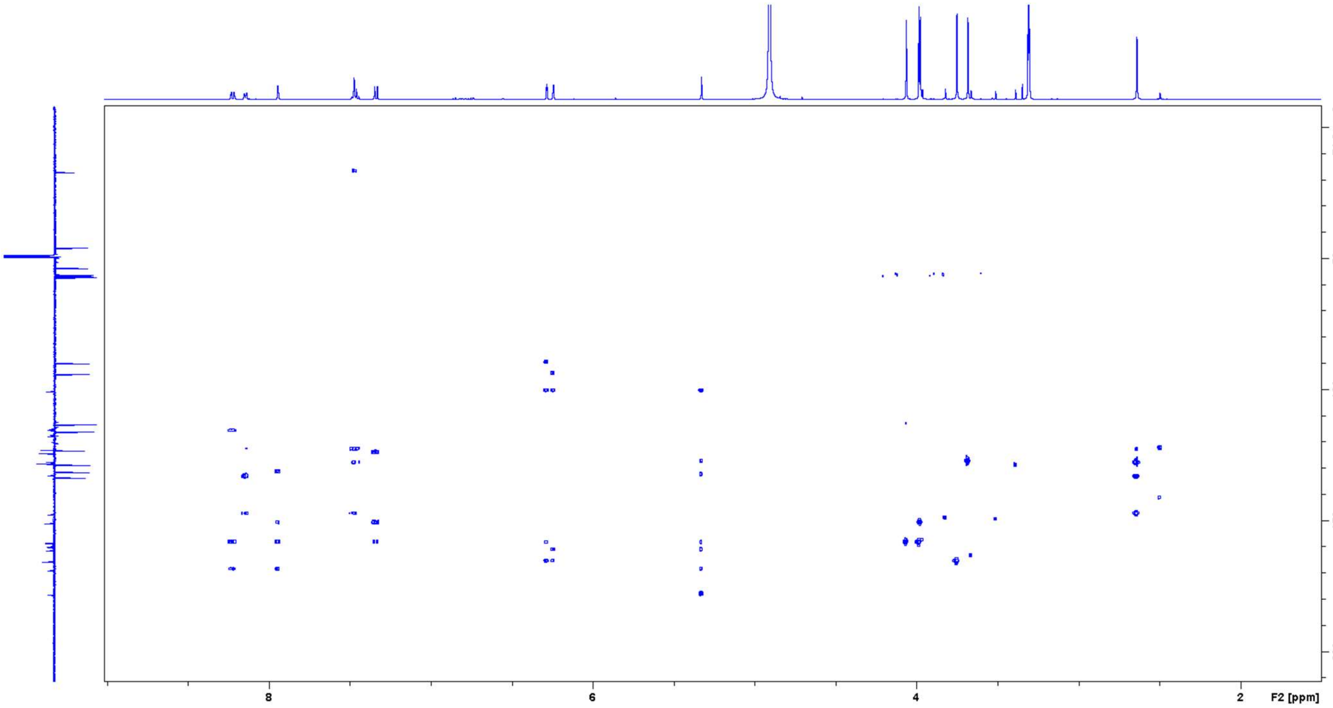

Figure S19: 18-Methyl-5,7,11,3‘,4‘-penta-*O*-methylnudicaulin (**9**), ^{1}H-^{13}C HMBC spectrum (500 MHz, 1% TFA - MeOH-d_4)

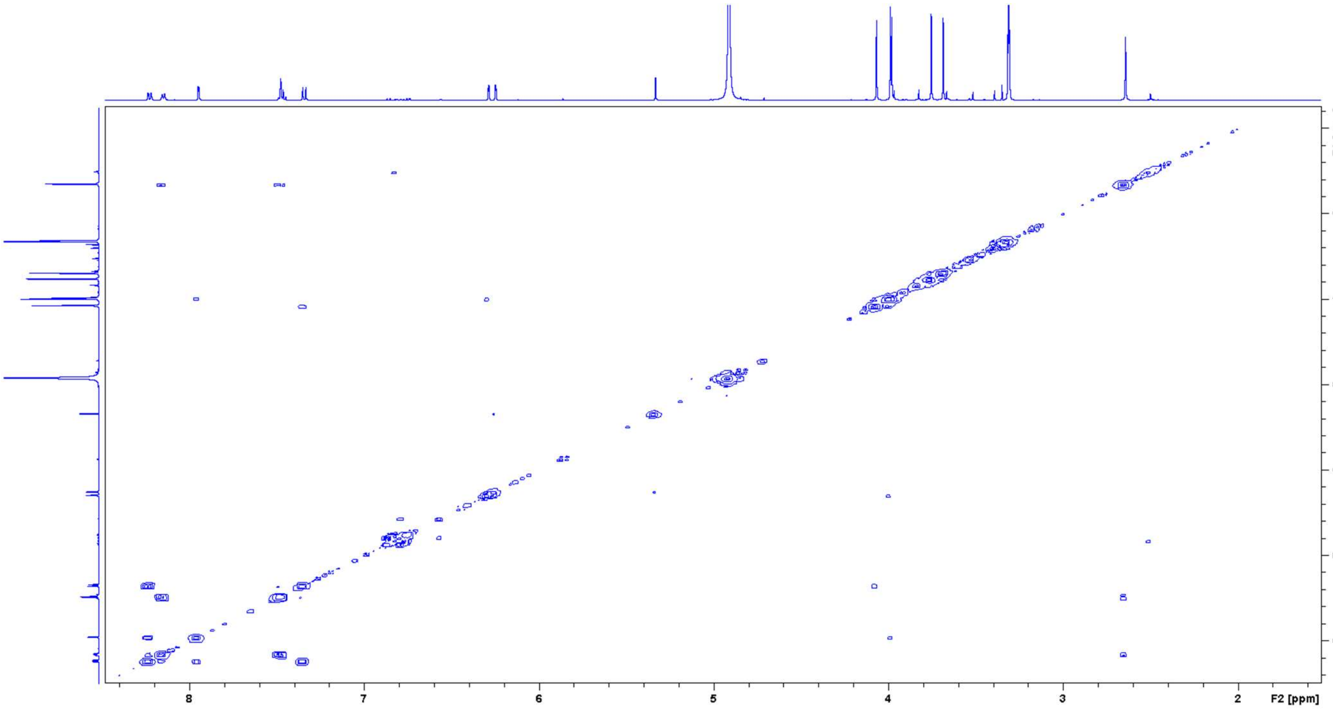

Figure S20: 18-Methyl-5,7,11,3‘,4‘-penta-*O*-methylnudicaulin (**9**), ^{1}H-^{1}H COSY spectrum (500 MHz, 1% TFA - MeOH-d_4)

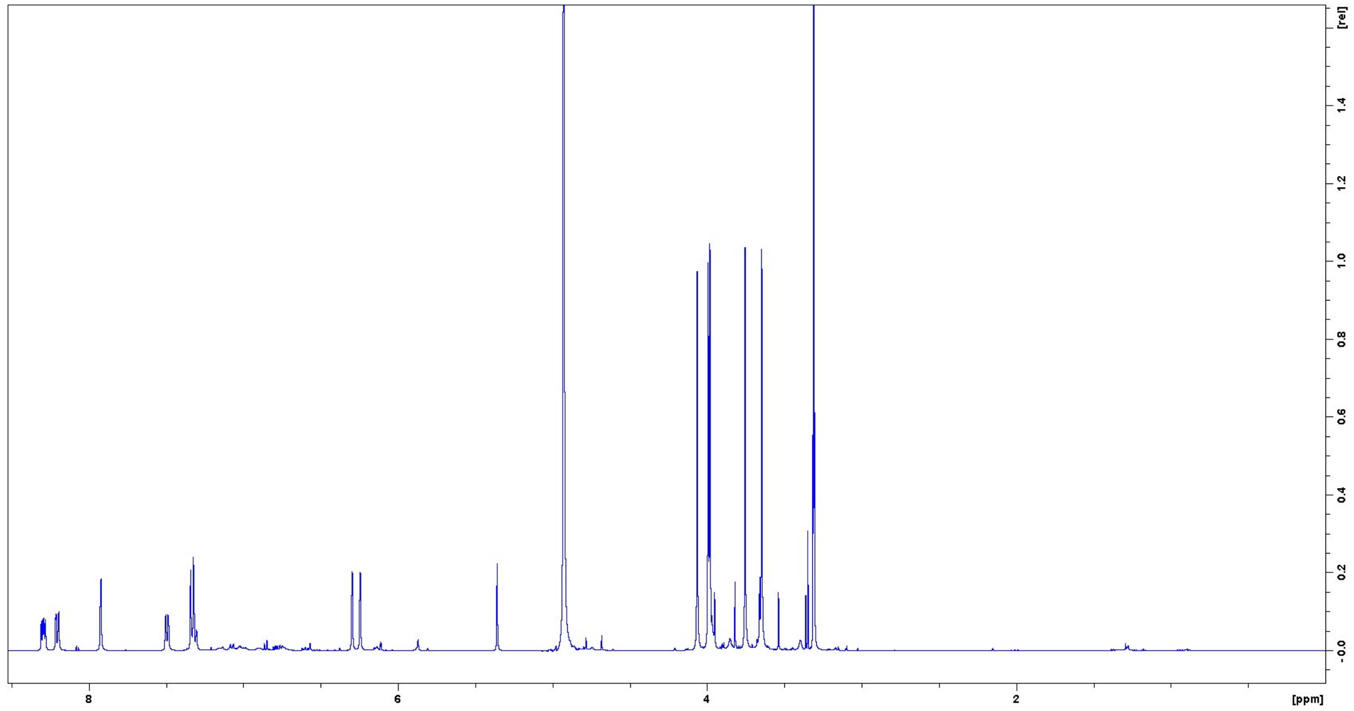

Figure S21: 17-Fluoro-5,7,11,3‘,4‘-penta-*O*-methylnudicaulin (**10**), ^{1}H-NMR spectrum (500 MHz, 1% TFA - MeOH-d_4)

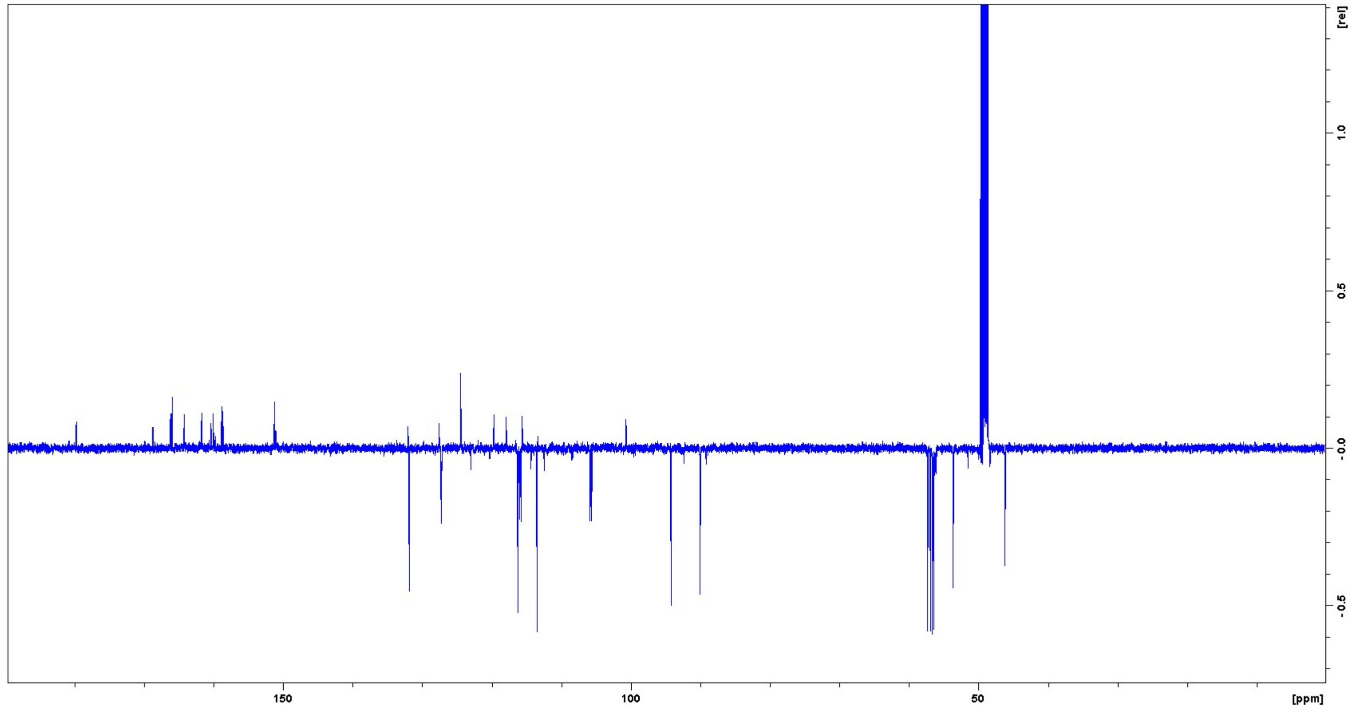

Figure S22: 17-Fluoro-5,7,11,3‘,4‘-penta-*O*-methylnudicaulin (**8**), ^{13}C-NMR spectrum (500 MHz, 1% TFA - MeOH-d_4)

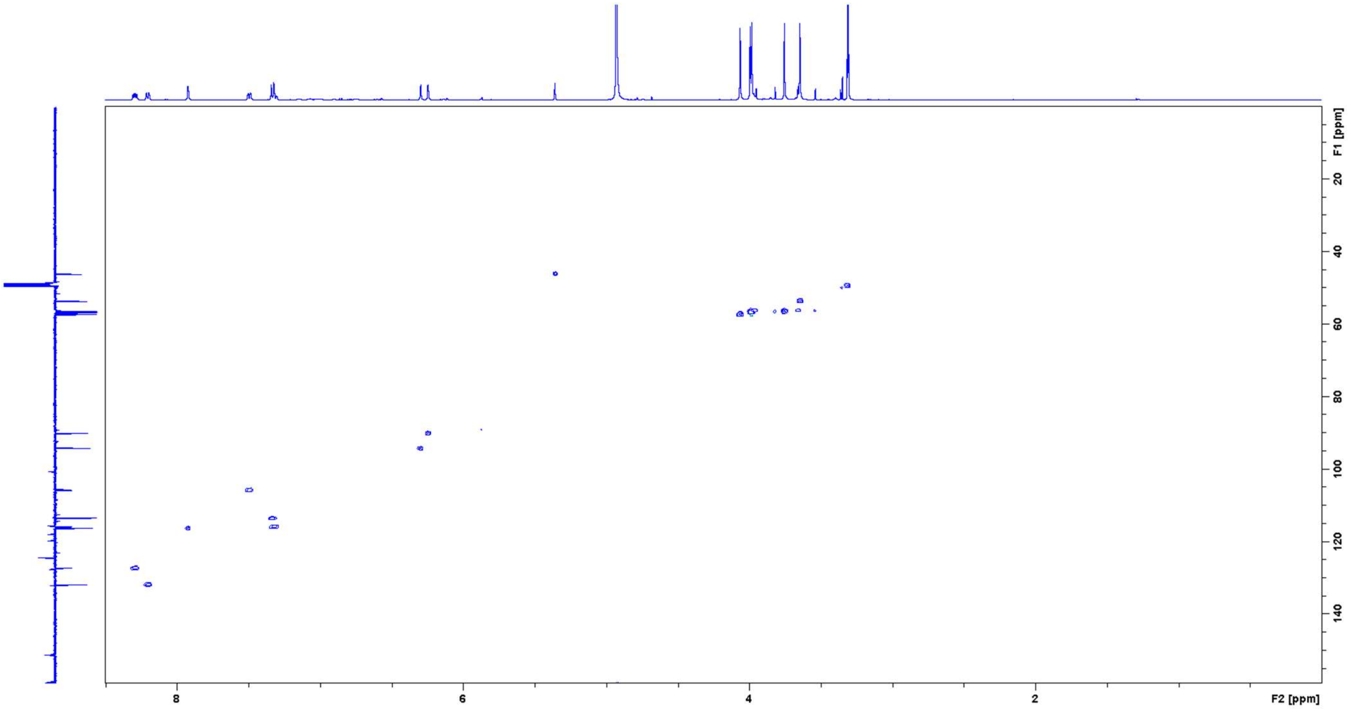

Figure S23: 17-Fluoro-5,7,11,3‘,4‘-penta-*O*-methylnudicaulin (**8**), ^{1}H-^{13}C HSQC spectrum (500 MHz, 1% TFA - MeOH-d_4)

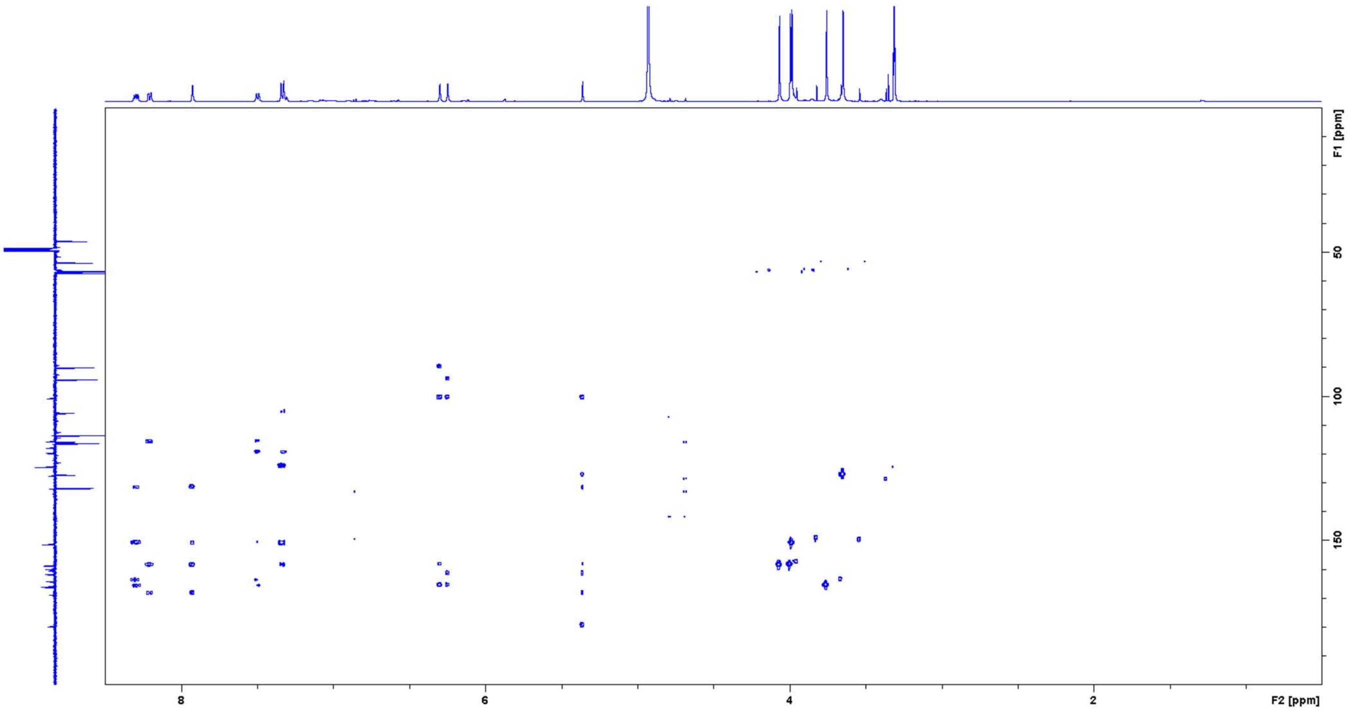

Figure S24: 17-Fluoro-5,7,11,3‘,4‘-penta-*O*-methylnudicaulin (**8**), ^{1}H-^{13}C HMBC spectrum (500 MHz, 1% TFA - MeOH-d_4)

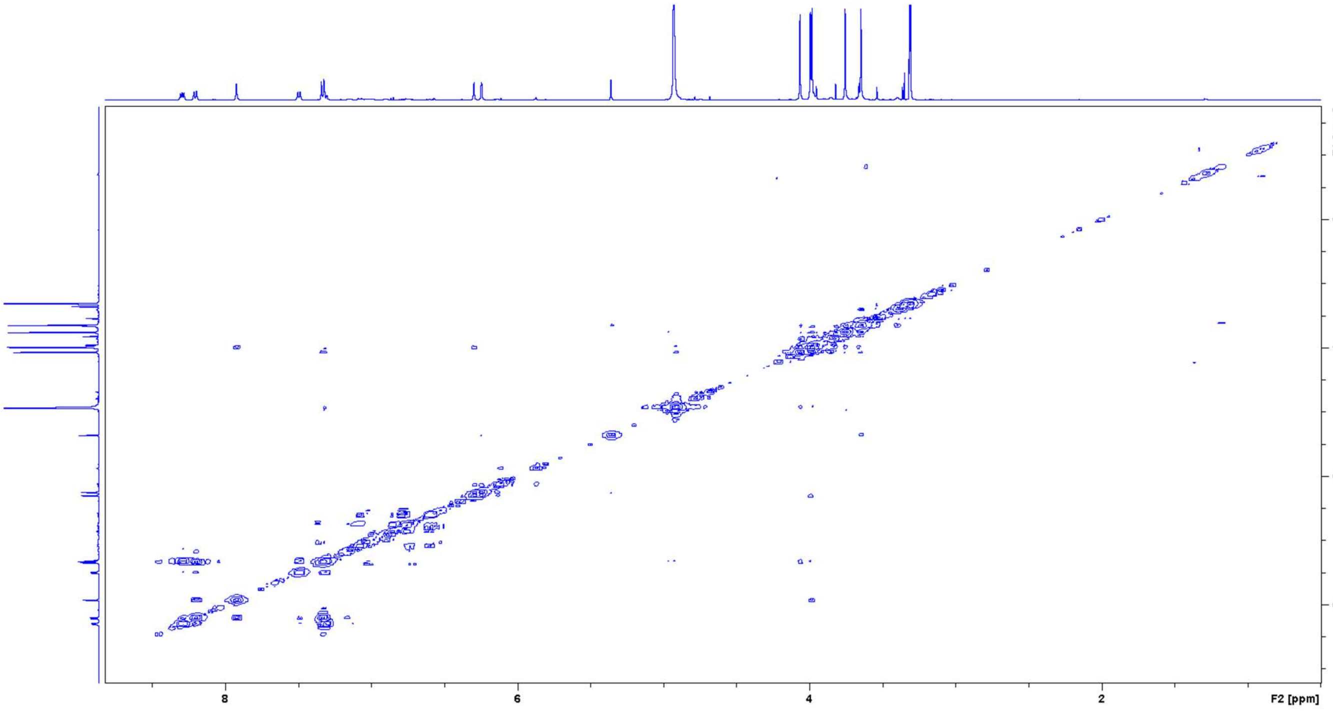

Figure S25: 17-Fluoro-5,7,11,3‘,4‘-penta-*O*-methylnudicaulin (**10**), ^{1}H-^{1}H COSY spectrum (500 MHz, 1% TFA - MeOH-d_4)

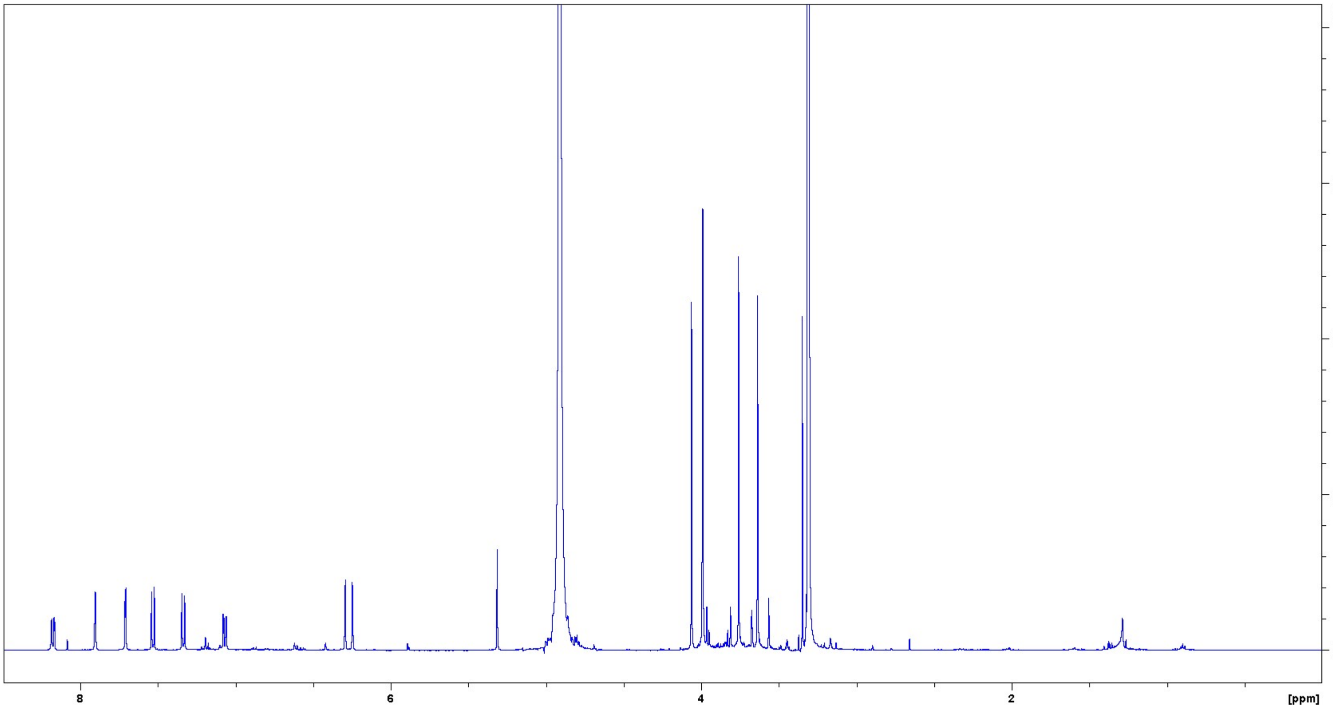

Figure S26: 17-Hydroxy-5,7,11,3‘,4‘-penta-*O*-methylnudicaulin (**11**), ^{1}H-NMR spectrum (500 MHz, 1% TFA - MeOH-d_4)

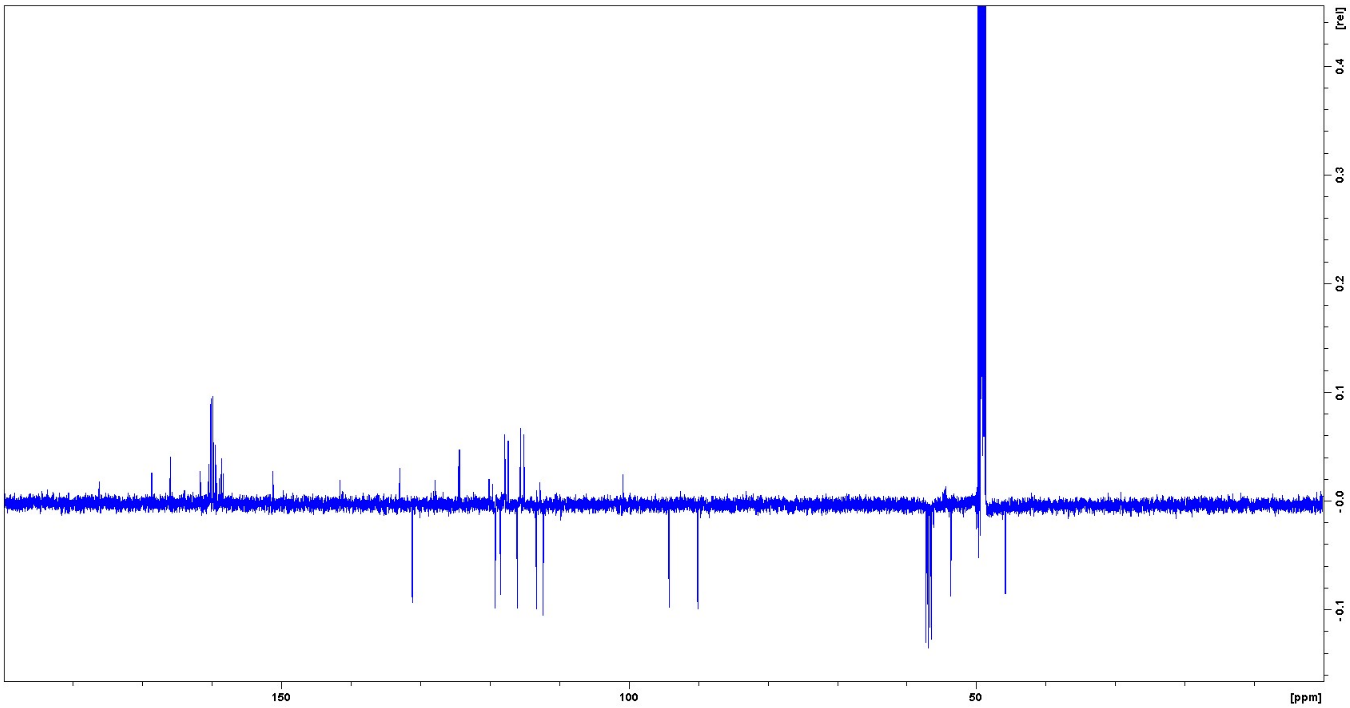

Figure S27: 17-Hydroxy-5,7,11,3‘,4‘-penta-*O*-methylnudicaulin (**11**), ^{13}C-NMR spectrum (500 MHz, 1% TFA - MeOH-d_4)

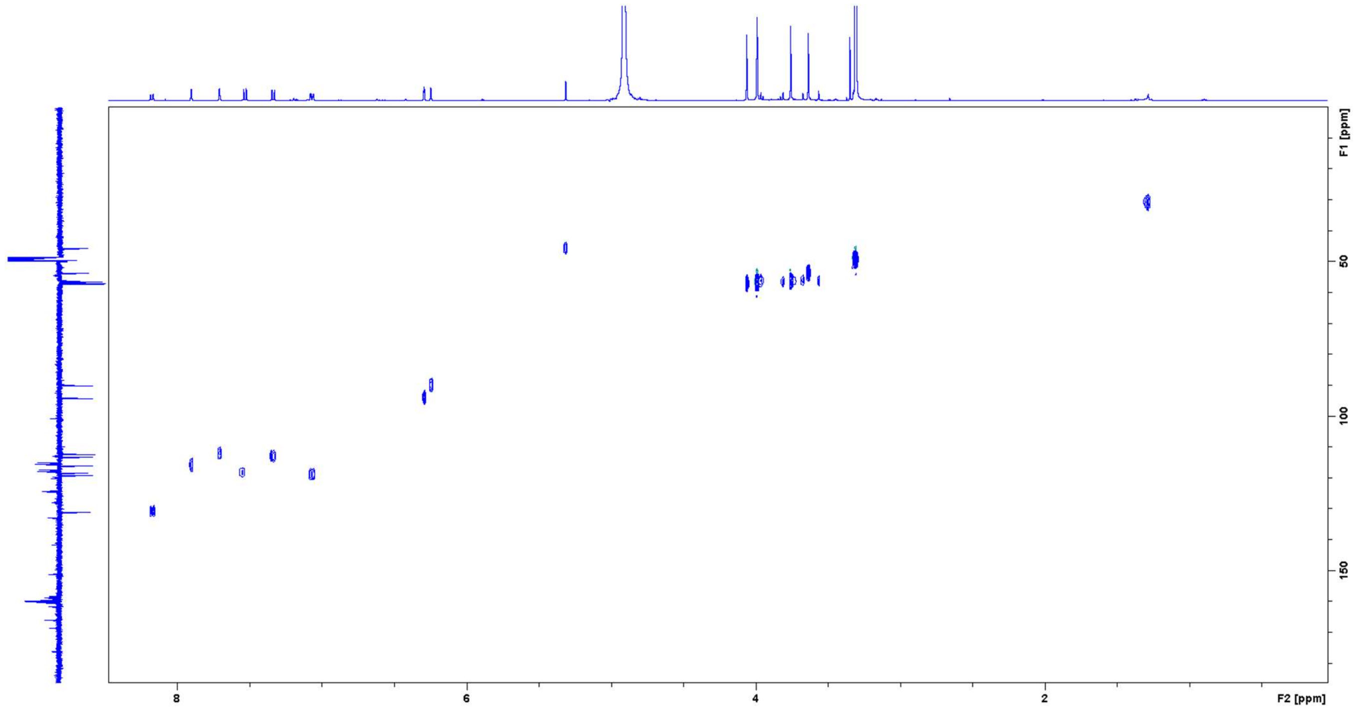

Figure S28: 17-Hydroxy-5,7,11,3‘,4‘-penta-*O*-methylnudicaulin (**11**), ^{1}H-^{13}C HSQC spectrum (500 MHz, 1% TFA - MeOH-d_4)

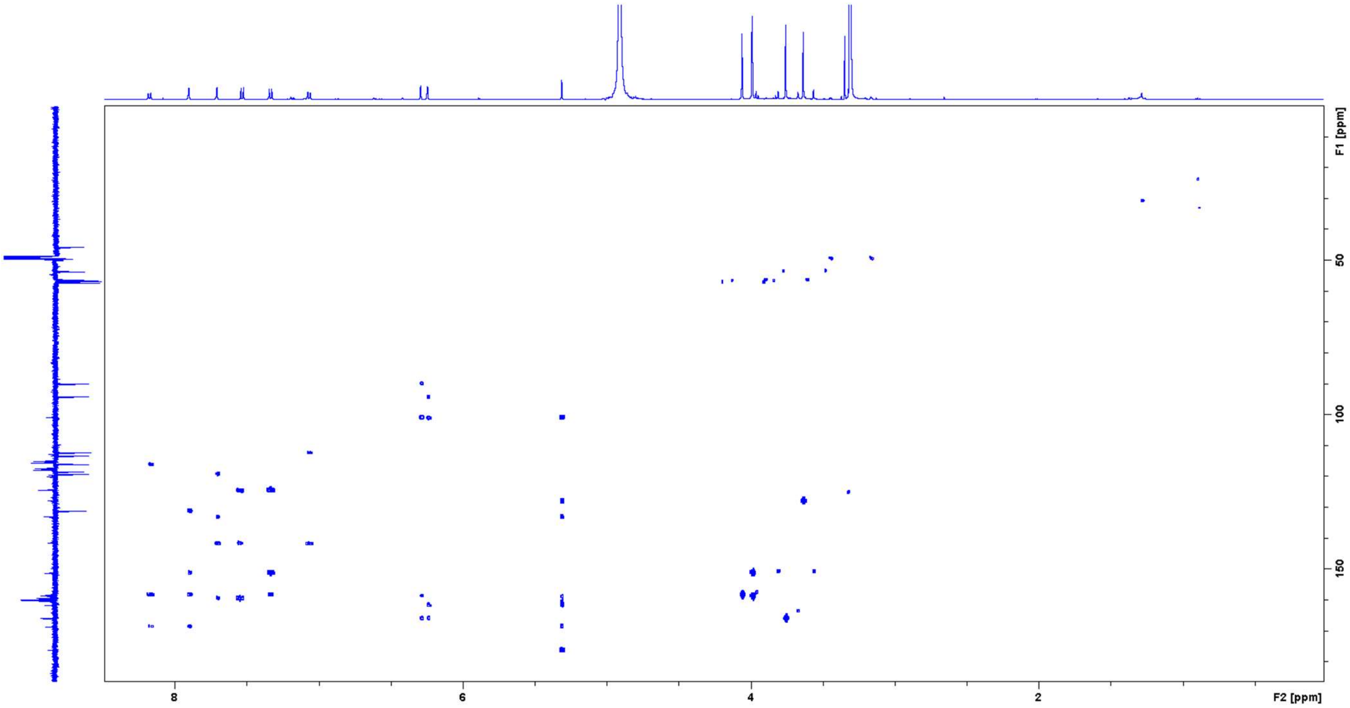

Figure S29: 17-Hydroxy-5,7,11,3‘,4‘-penta-*O*-methylnudicaulin (**11**), ^{1}H-^{13}C HMBC spectrum (500 MHz, 1% TFA - MeOH-d_4)

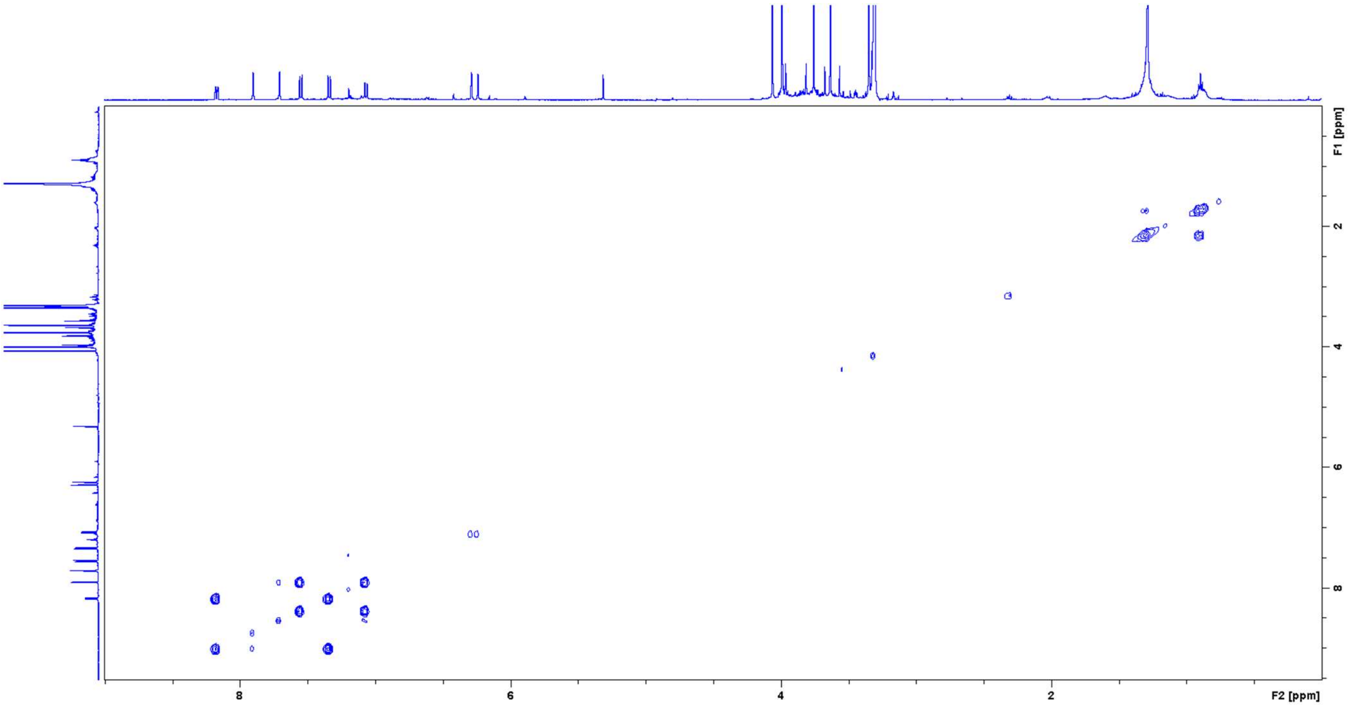

Figure S30: 17-Hydroxy-5,7,11,3‘,4‘-penta-*O*-methylnudicaulin (**10**), ^{1}H-^{1}H COSY spectrum (500 MHz, 1% - MeOH-d_4)

3. HR-MS

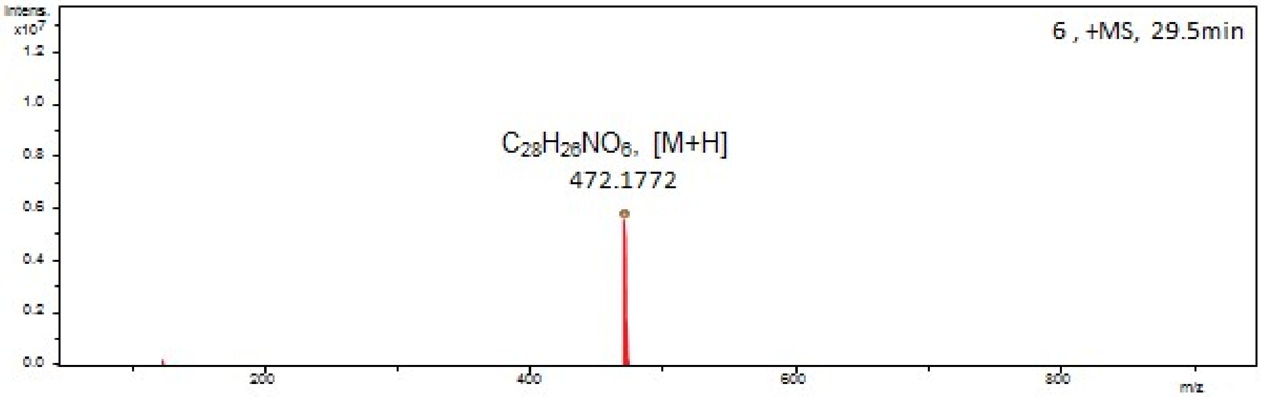

Figure S31: 5,7,11,3‘,4‘-Penta-*O*-methylnudicaulin (**6**), mass spectrum

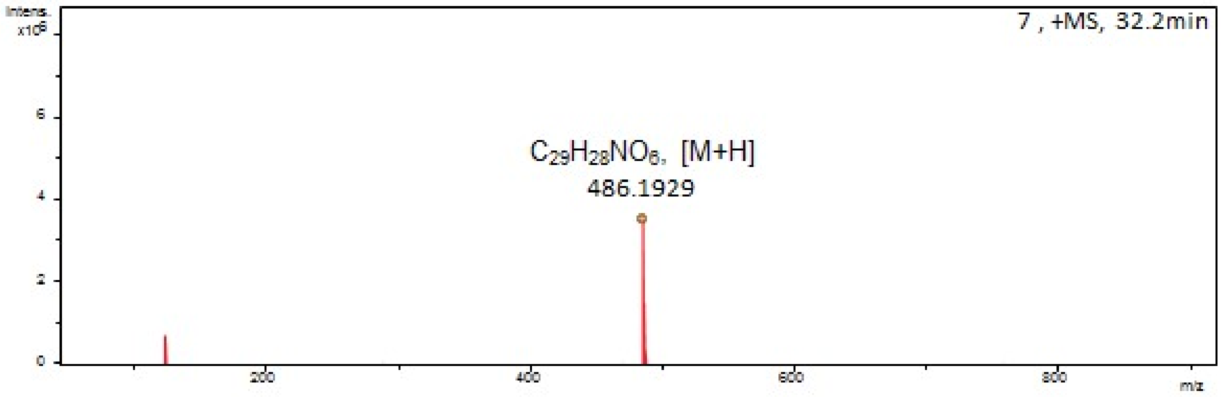

Figure S32: 16-Methyl-5,7,11,3‘,4‘-penta-*O*-methylnudicaulin (**7**), mass spectrum

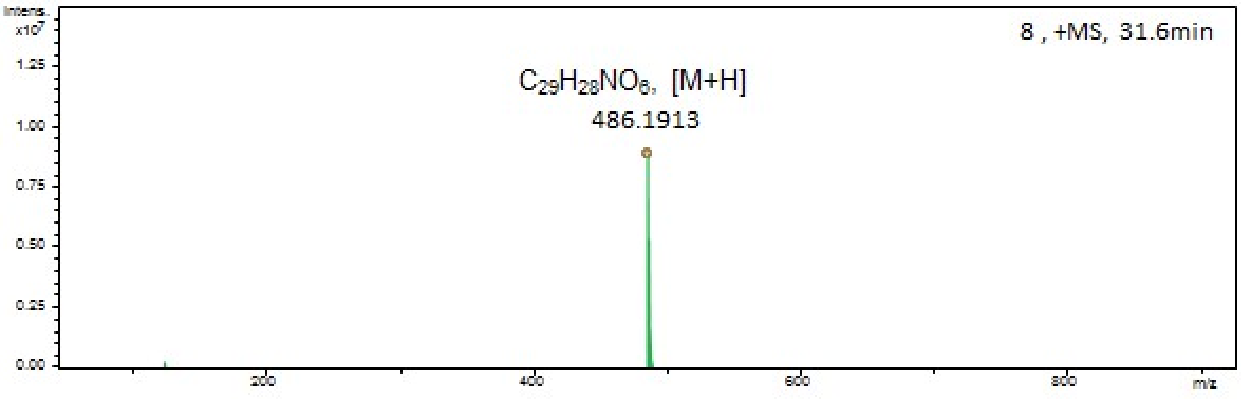

Figure S33: 17-Methyl-5,7,11,3‘,4‘-penta-*O*-methylnudicaulin (**8**), mass spectrum

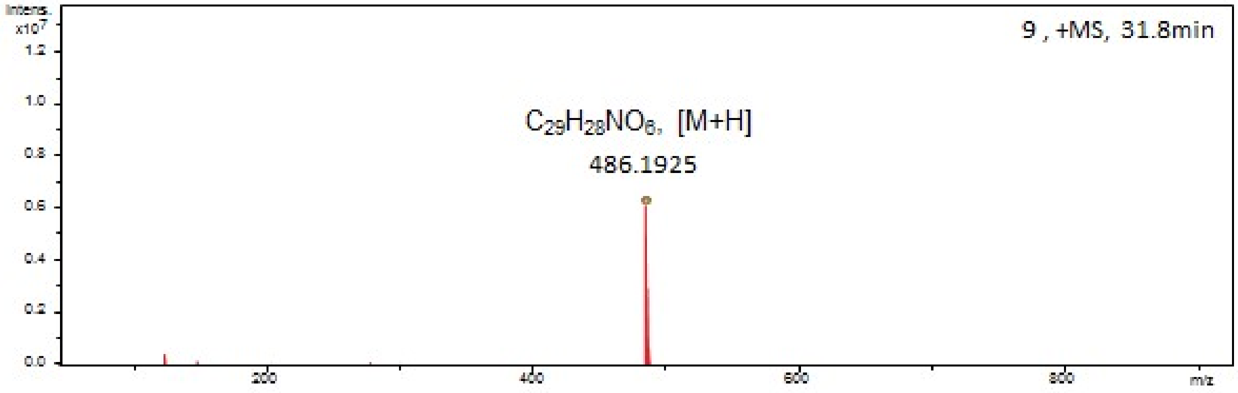

Figure S34: 18-Methyl-5,7,11,3‘,4‘-penta-*O*-methylnudicaulin (**9**), mass spectrum

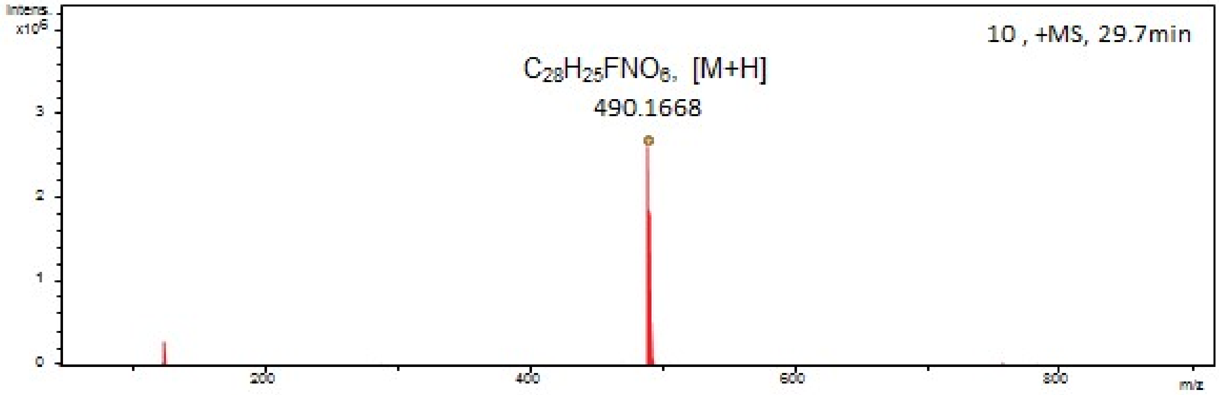

Figure S35: 17-Fluoro-5,7,11,3‘,4‘-penta-*O*-methylnudicaulin (**10**), mass spectrum

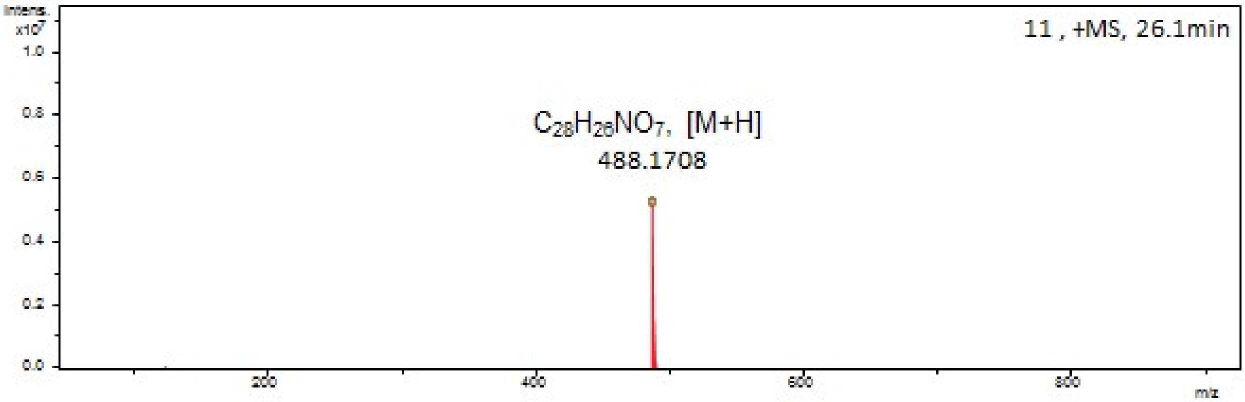

Figure S36: 17-Hydroxy-5,7,11,3‘,4‘-penta-*O*-methylnudicaulin (**11**), mass spectrum

4. UV/Vis absorption spectra

UV/Vis absorption spectra were acquired with the photodiode array detector (PDA) G1315B, 200-700 nm of *HPLC 1* (Chapter **4.1**).

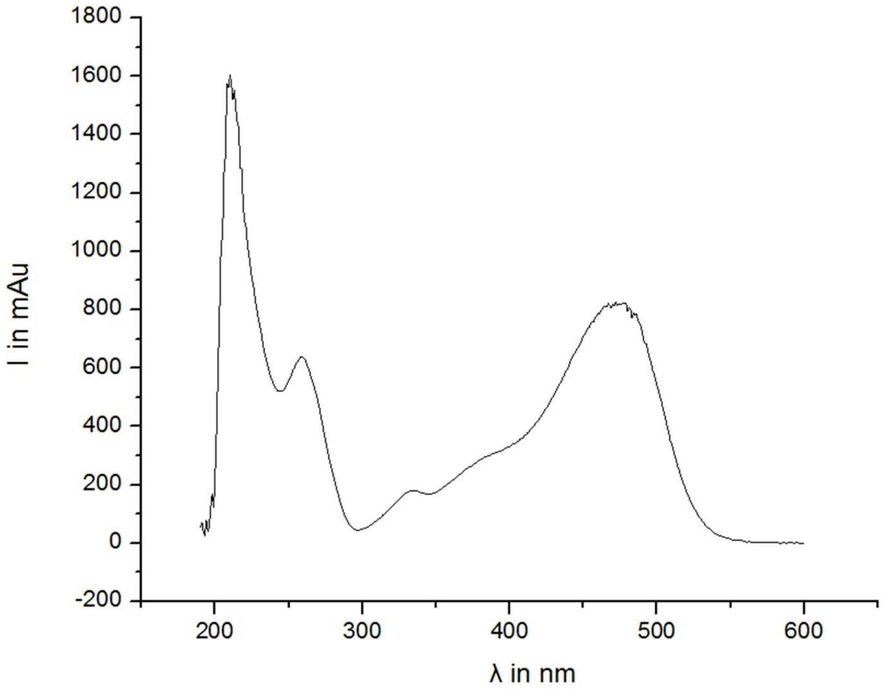

Figure S37: 5,7,11,3‘,4‘-Penta-*O*-methylnudicaulin (**6**), UV/Vis spectrum (0.1 % TFA-MeOH)

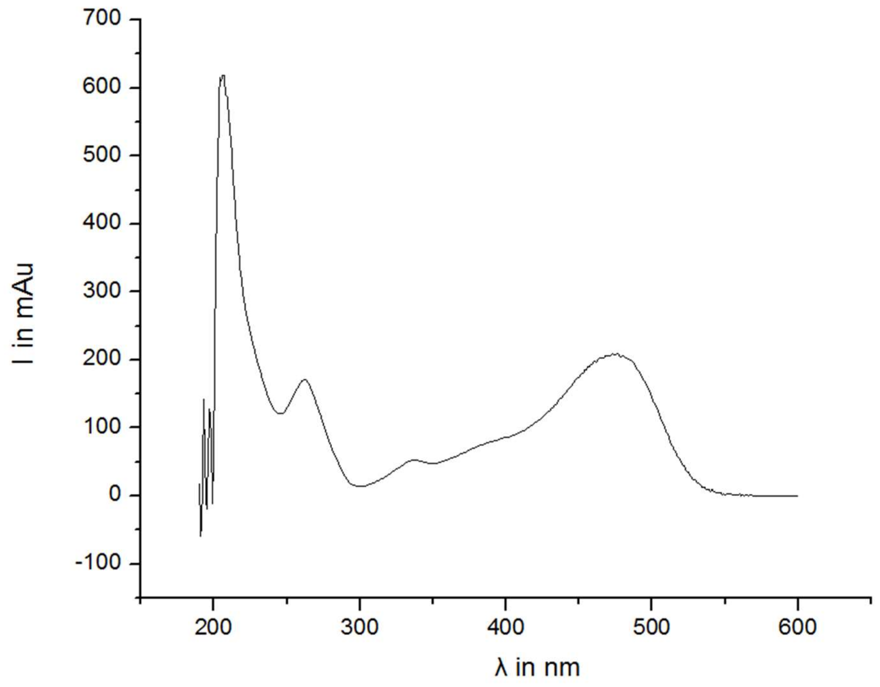

Figure S38: 16-Methyl-5,7,11,3‘,4‘-penta-*O*-methylnudicaulin (**7**), UV/Vis spectrum (0.1 % TFA-MeOH)

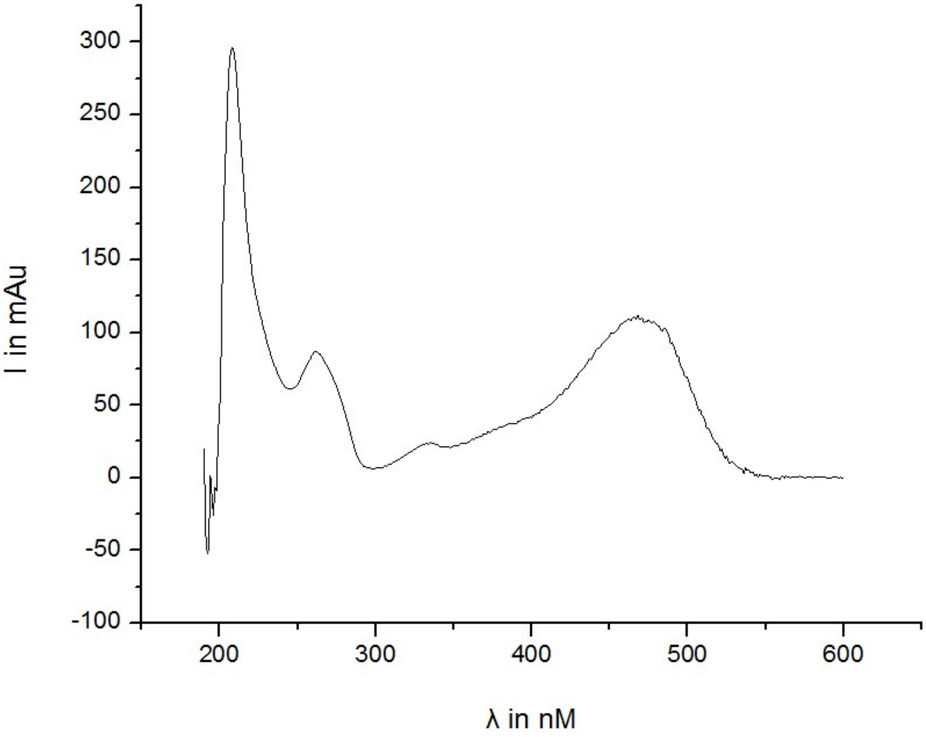

Figure S39: 17-Methyl-5,7,11,3‘,4‘-penta-*O*-methylnudicaulin (**8**), UV/Vis spectrum (0.1 % TFA-MeOH)

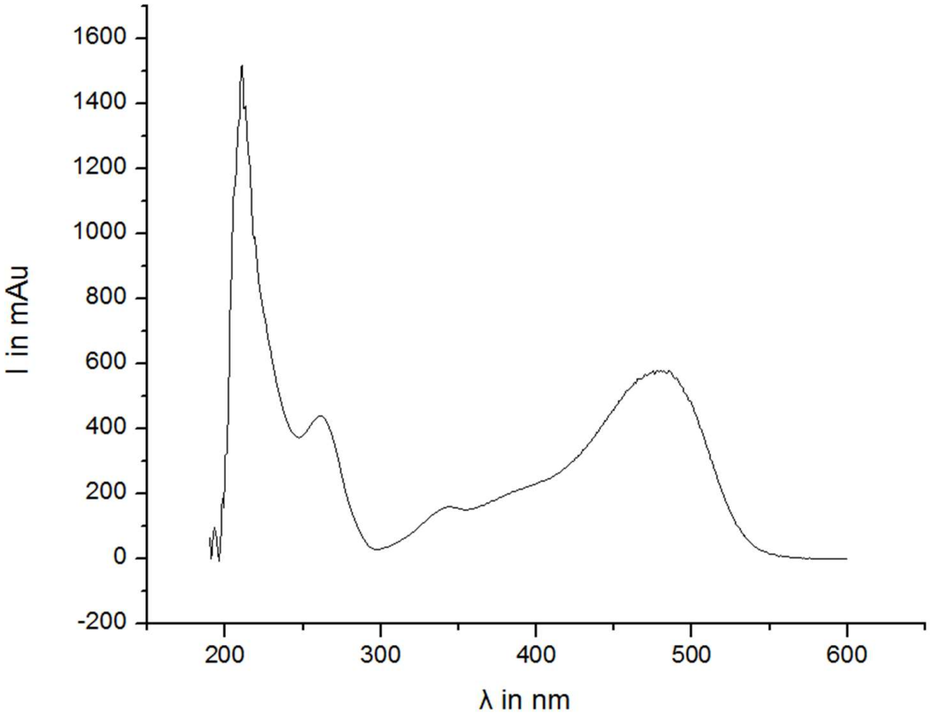

Figure S40: 18-Methyl-5,7,11,3‘,4‘-penta-*O*-methylnudicaulin (**9**), UV/Vis spectrum (0.1 % TFA-MeOH)

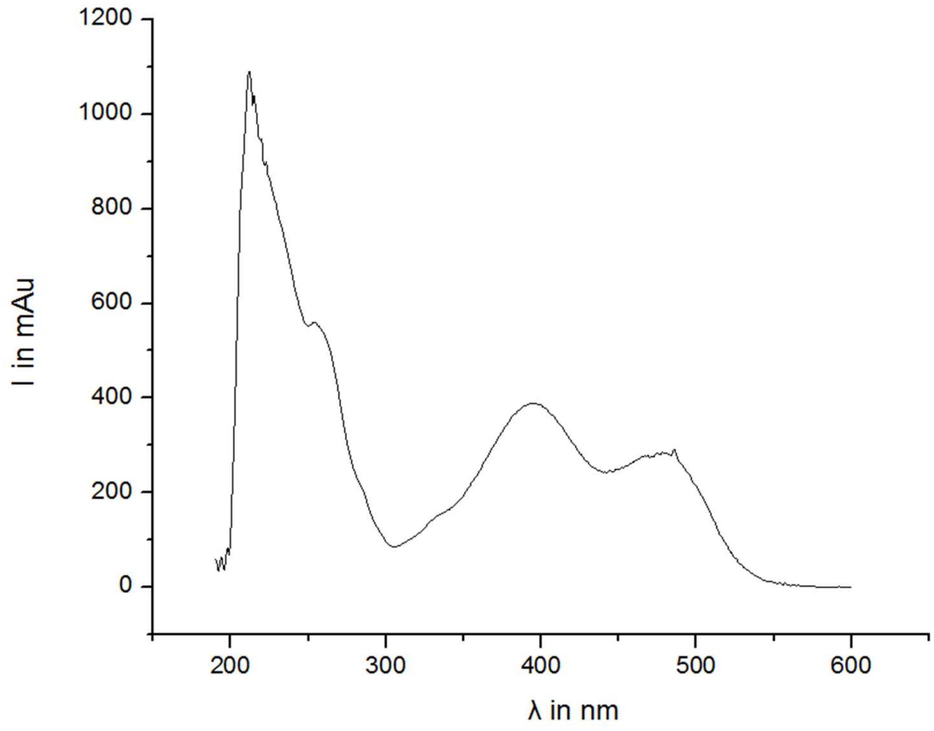

Figure S41: 17-Fluoro-5,7,11,3‘,4‘-penta-*O*-methylnudicaulin (**10**), UV/Vis spectrum (0.1 % TFA-MeOH)

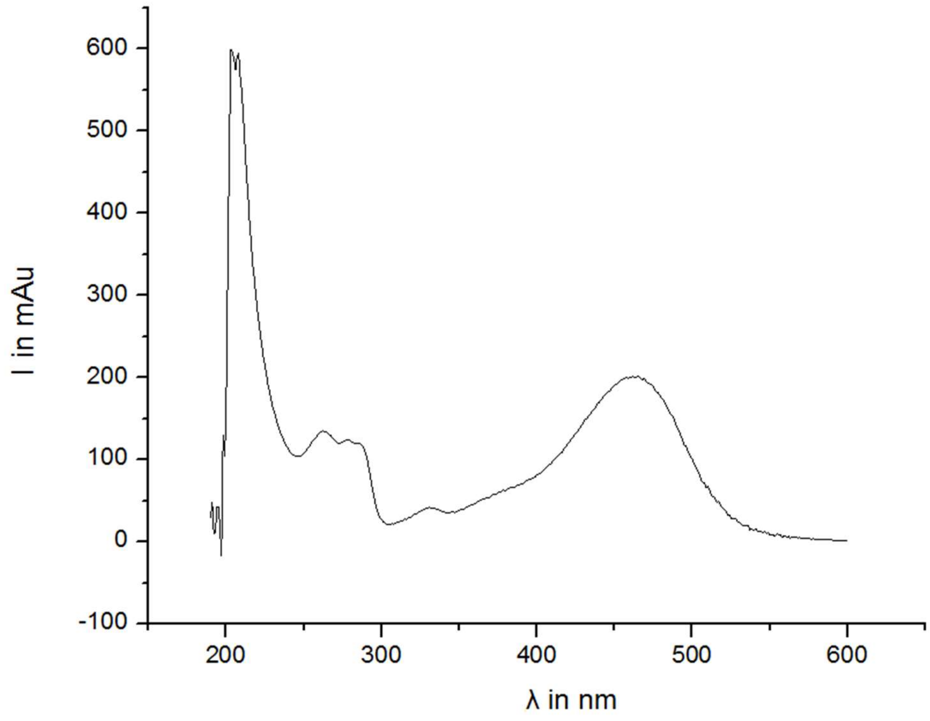

Figure S42: 17-Hydroxy-5,7,11,3‘,4‘-penta-*O*-methylnudicaulin (**11**), UV/Vis spectrum (0.1 % TFA-MeOH)

5. CD spectrum

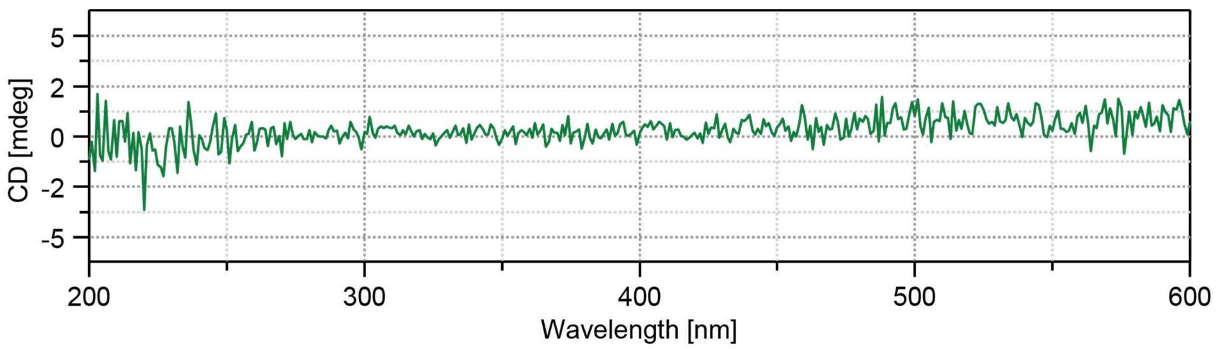

Figure S43: 5,7,11,3‘,4‘-Penta-*O*-methylnudicaulin (**6**), CD spectrum (concentration 0.78 mg ml^{-1} (1.66 mM in MeOH), cuvette width 1 mm)

6. Cell Toxicity

*Table S1: Antiproliferative and cytotoxicity data of compounds **6**-**11***

	Antiproliferative activity		Cytotoxicity
Compound	HUVEC GI_{50} [µmol l^{-1}]	K-562 GI_{50} [µmol l^{-1}]	HeLa CC_{50} [µmol l^{-1}]
**6**	1.3 (± 0.2)	1.1 (± 0.4)	3.4 (± 0.4)
**7**	2.5 (± 1.2)	1.4 (± 0.6)	5.8 (± 0.2)
**8**	5.3 (± 1.9)	4.1 (± 0.6)	16.5 (± 1.4)
**9**	2.9 (± 0.4)	2.9 (± 0.4)	8.4 (± 1.2)
**10**	2.2 (± 0.6)	1.0 (± 0.2)	5.7 (± 0.2)
**11**	2.0 (± 0.4)	1.0 (± 0.4)	5.5 (± 0.2)
**Imatinib***	22.1 (± 2.4)	0.2 (± 1.4×10^{-2})	78.6 (± 2.8)
**Doxorubicin***	0.2	1.0 (± 1.1)	3.68 (± 1.5)

For structures of compounds **6**-**11**, see Scheme II.

* Chemotherapeutic drugs, values published in [23] Krauth, F.; Dahse, H.-M.; Rüttinger, H.-H.; Frohberg, P. Synthesis and characterization of novel 1,2,4-triazine derivatives with antiproliferative activity. *Bioorg. Med. Chem.* **2010**, *18*, 1816–1821, DOI 10.1016/j.bmc.2010.01.053.

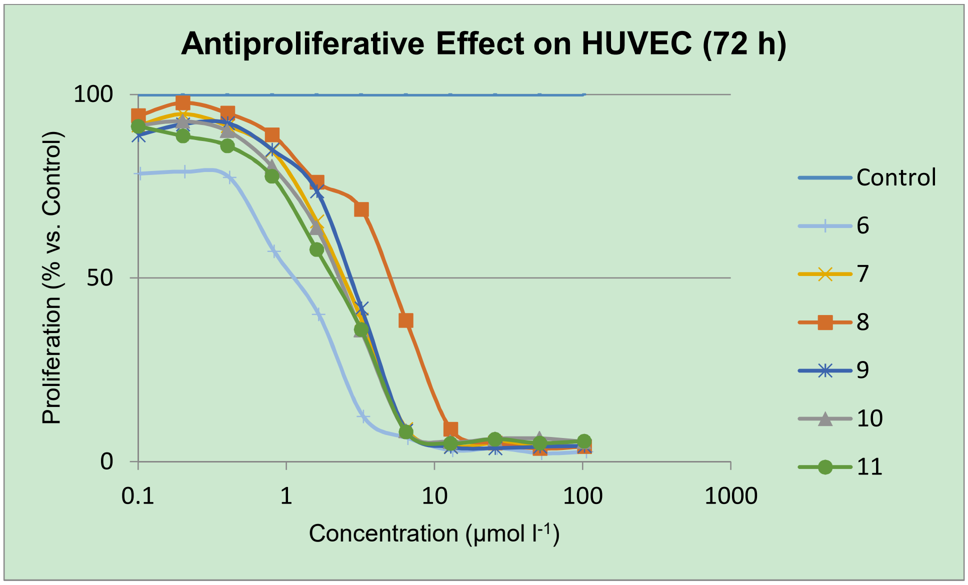

Figure S44: Dose–response relationship for antiproliferative effect on HUVEC cells (72 h), Representative curves are given.

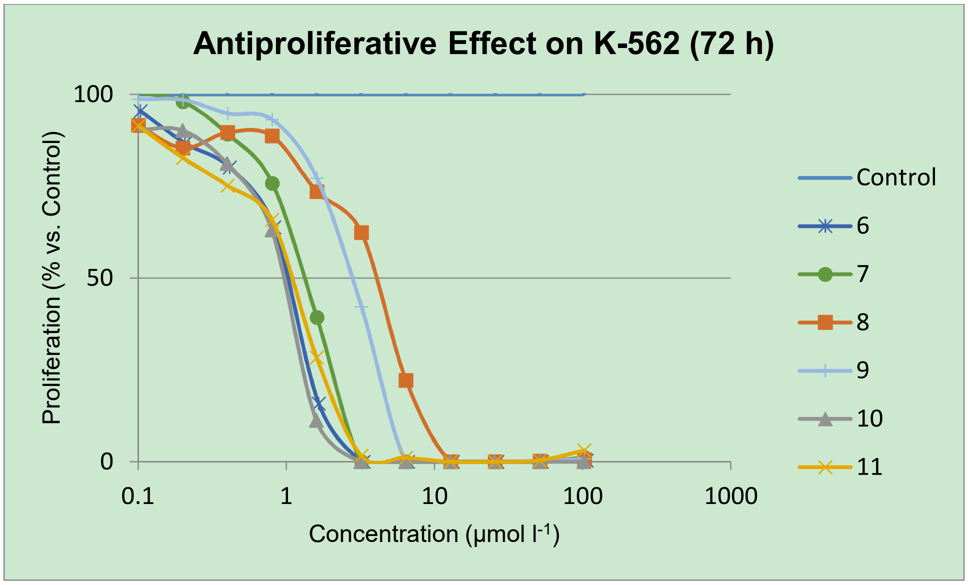

Figure S45: Dose–response relationship values for antiproliferative effect on K-562 cells (72 h), Representative curves are given.

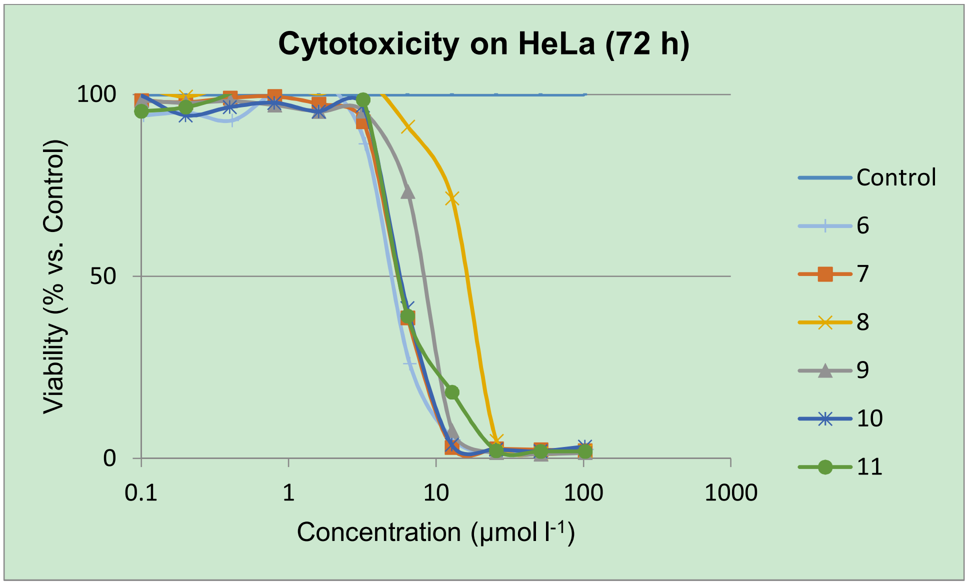

Figure S46: Dose–response relationship values for cytotoxicity on HeLa cells (72 h), Representative curves are given.

7. Antimicrobial activity

*Table S2: Antimicrobial data of compounds **6**-**11***

Compound		6*	7*	8*	9*	10*	11*	Ciprofla-xacin**	MeOH	Amphote-ricin B***
c [mg ml^{-1}]		1	1	1	1	1	1	5*10^{-3}	0	1*10^{-2}
Bacillus subtilis	6633 B1	0	0	12P	0	0	0	28	0	0
Staphylococcus aureus	511 B3	0	0	13P	0	0	0	18	0	0
Escherichia coli	458 B4	0	0	0	0	0	0	24/ 32p	0	0
Pseudomonas aeruginosa	SG137 B7	0	0	0	0	0	0	26	10	0
P. aeruginosa	K799/61 B9	0	0	0	0	0	0	28/37p	0	0
MRSA S. aureus	134/93 R9	0	0	13P	0	0	0	0	0	0
VRE Enterococcus faecalis	1528 R10	0	0	0	0	0	0	17F	0	0
Mycobacterium vaccae	10670 M4	18p	14p	14p	15p	15p	18p	23p	0	0
Sporobolomyces salmonicolor	549 H4	0	0	0	0	0	0	0		18p
Candida albicans	C. alb H8	0	0	0	0	0	0	0	0	21
Penicillium notatum	JP36 P1	0	0	0	0	0	0	0	0	18p

For structures of compounds **6**-**11**, see Scheme II.

p = colonies in the inhibition zone; P = many colonies in the inhibition zone; A = indication of inhibition; F = promotion of growth

* solution in MeOH

** solution in deionized water

*** solution in DMSO/MeOH

MRSA indicates methicillin-resistant *Staphylococcus aureus*

VRE indicates vancomycin-resistant *Enterococcus*
